# Cell-Type-Specific Predictive Network Yields Novel Insights into Mouse Embryonic Stem Cell Self-Renewal and Cell Fate

**DOI:** 10.1371/journal.pone.0056810

**Published:** 2013-02-28

**Authors:** Karen G. Dowell, Allen K. Simons, Zack Z. Wang, Kyuson Yun, Matthew A. Hibbs

**Affiliations:** 1 The Jackson Laboratory, Bar Harbor, Maine, United States of America; 2 Graduate School of Biomedical Sciences and Engineering, University of Maine, Orono, Maine, United States of America; 3 Johns Hopkins University, Baltimore, Maryland, United States of America; 4 Trinity University, Department of Computer Science, San Antonio, Texas, United States of America; Queen’s University Belfast, United Kingdom

## Abstract

Self-renewal, the ability of a stem cell to divide repeatedly while maintaining an undifferentiated state, is a defining characteristic of all stem cells. Here, we clarify the molecular foundations of mouse embryonic stem cell (mESC) self-renewal by applying a proven Bayesian network machine learning approach to integrate high-throughput data for protein function discovery. By focusing on a single stem-cell system, at a specific developmental stage, within the context of well-defined biological processes known to be active in that cell type, we produce a consensus predictive network that reflects biological reality more closely than those made by prior efforts using more generalized, context-independent methods. In addition, we show how machine learning efforts may be misled if the tissue specific role of mammalian proteins is not defined in the training set and circumscribed in the evidential data. For this study, we assembled an extensive compendium of mESC data: ∼2.2 million data points, collected from 60 different studies, under 992 conditions. We then integrated these data into a consensus mESC functional relationship network focused on biological processes associated with embryonic stem cell self-renewal and cell fate determination. Computational evaluations, literature validation, and analyses of predicted functional linkages show that our results are highly accurate and biologically relevant. Our mESC network predicts many novel players involved in self-renewal and serves as the foundation for future pluripotent stem cell studies. This network can be used by stem cell researchers (at http://StemSight.org) to explore hypotheses about gene function in the context of self-renewal and to prioritize genes of interest for experimental validation.

## Introduction

Stem cells, uniquely characterized by their ability to self-renew and differentiate, are a promising tool for biomedical research and cell-based therapy. These special cells play pivotal roles in many stages of normal organism development as well as tissue homeostasis and repair [1]. The properties of “stemness” have also been observed in unnaturally stem-like cells, including artificially induced pluripotent stem (iPS) cells, immortalized cell lines, and cancers [2]–[4]. A comprehensive, systems-level view of pluripotent cell self-renewal processes will not only advance our knowledge of stem cell biology, but also facilitate the development of safer biomedical applications.

During development, gene expression profiles change continuously as stem cells rapidly proliferate, differentiate, and communicate with each other. Terminally differentiated cells have more stable gene expression profiles that reflect their distinct roles within tissues and organs; the molecular composition of these mature cells differs dramatically depending on cellular function [5]. To manage complexity and minimize confounding factors, most mammalian laboratory experiments are limited to a specific cell type, tissue, or system of interest [6]. However, most bioinformatics and systems biology approaches have not yet addressed cell- and tissue-specific concerns.

### Computational Methods for Predicting Protein Function Using High-throughput Data

Machine learning techniques based on high-throughput data integration have been used to predict protein function in mammals with mixed results [7], [8]. Naïve Bayesian networks (Bayes nets), one form of supervised machine learning, have proven successful for gene function discovery as they provide a statistically principled method to model relationships among proteins based on a solid foundation of biological knowledge [7], [9]–[13]. Given a training set of prior knowledge (also known as a gold standard) comprised of protein pairs known to be functionally related (positive training examples) and pairs believed to be unrelated (negative training examples) combined with independent, whole-genome high-throughput datasets (observed evidential data), Bayes nets identify significant patterns in the evidence, assess data reliability, and then predict novel protein relationships based on reliable data [14], [15]. This method is straightforward and facilitates precise control over gold standard and evidential data composition for testing.

Using Bayes net methodologies originally developed for yeast [14], [15], several studies have predicted mammalian protein function by treating mammals as homogenous, single-celled organisms [16]–[24]. These studies were species-specific and focused on integrating diverse high-throughput data from multiple cell types and tissues. They leveraged functional annotations provided by public resources, such as the Gene Ontology (GO) and the Kyoto Encyclopedia of Genes and Genomes (KEGG) [25], [26], to automatically generate training sets. Results of these pioneering efforts showed that protein function can be predicted accurately using a generalized methodology [17], [18], [20], [27]. However, due to the current state of public annotation resources, these methods are not appropriate for predicting cell-type-specific protein function in mammalian systems. This is because, historically, if a protein performs a function in any cellular or *in vitro* context, it was annotated to that function. For example, the Mouse Genome Informatics (MGI) database referenced 68 GO term annotations for *Stat3*, a protein expressed in more than 174 mouse tissues (as of September 2012) [28]. (Appendix S1 lists symbols, names, and synonyms for all genes mentioned and includes references for abbreviations used in this article.) In mESCs, *Stat3* is a regulator of self-renewal through the LIF-induced JAK/STAT signaling pathway; in hepatocytes, it is involved in many physiological processes, from liver regeneration to apoptosis to metabolism [28]–[30]. *Stat3* has also been associated with abnormal temperature homeostasis, eating behavior, sexual reproduction, and other phenotypes [28]. The function of *Stat3* is likely to be highly dependent on cofactors, signaling pathways, and other cellular states. Thus, machine learning methods can be misled if the tissue-specific role of mammalian proteins is not defined in the training set and circumscribed in the evidential data.

**Figure 1:**
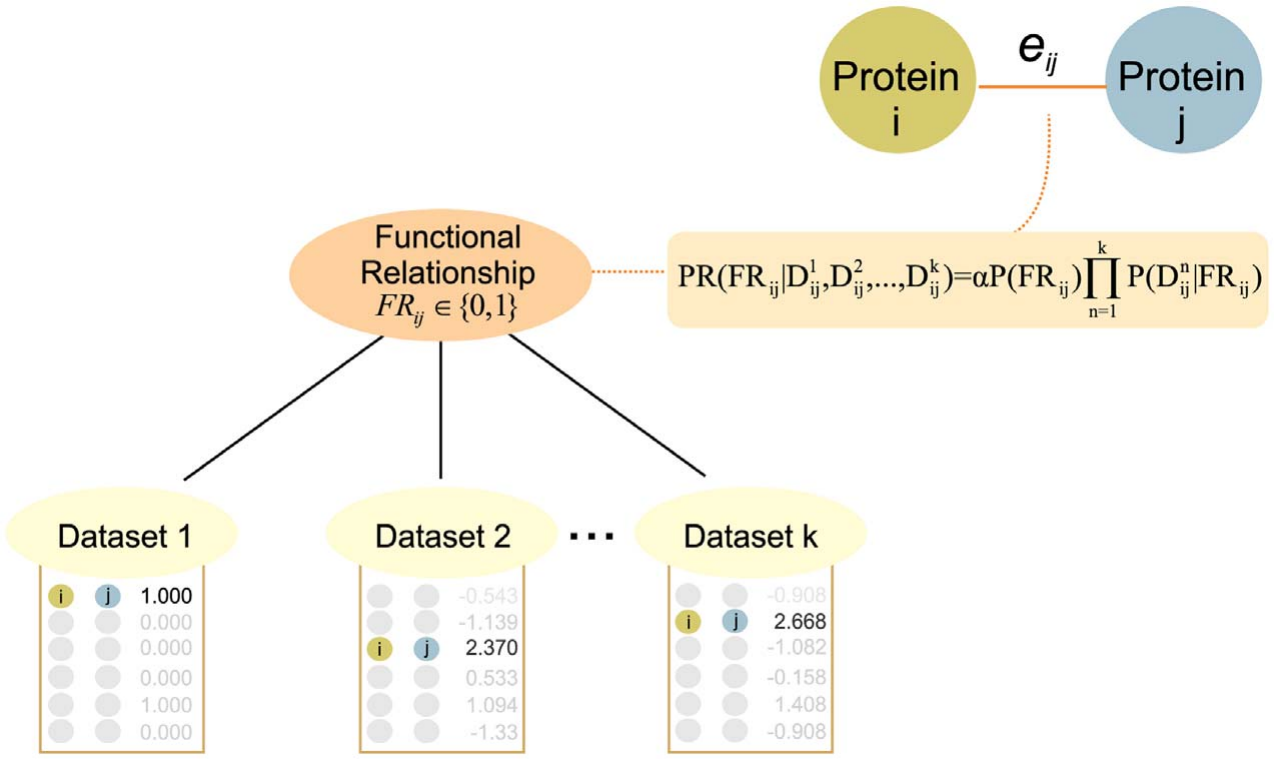
Naïve Bayesian Networks for Genomic Data Integration. A Bayesian network is a machine learning tool for organizing and encoding statistical dependence relationships among pieces of knowledge. A naïve Bayesian network is a simplified version of a Bayesian network in which all child nodes are dependent on the parent and independent of each other. This type of graphical device may be used to combine different types of evidential data and prior knowledge to generate probabilistic models of biological functional relationship networks. In our naïve Bayes net structure, the functional relationship between the pair of proteins *i* and *j* (FR_ij_) is a hidden conditional variable (indicating the unknown or hidden probability that these two gene products are functionally associated), on which all dataset evidence variables are dependent, and represents the discretized, observed similarity score in dataset *k* for proteins *i* and *j*. The edge weight (e_ij_) represents the probability that the proteins *ij* are functionally related given the evidence observed in different high-throughput datasets. Strong evidence of a functional relationship between protein pairs, measured by edge weight, indicates the proteins behave in a similar way given observed patterns in the high-throughput data. The specific nature of that relationship can be deduced by evaluating the type of datasets that contribute to that edge weight, followed experimental validation.

Here, we demonstrate the utility of predicting cell-type-specific protein function for mESCs and discuss the computational challenges of this task. Specifically, we show that Bayesian network integration methodologies (Figure 1) are most useful when applied to a focused biological question, such as a single cell type and biological processes known to be active in that cell.

mESCs are an attractive model system for testing cell-type-specific machine learning techniques because they are relatively homogenous, they have been extensively studied, and diverse high-throughput data collected from mESCs are publicly available. Despite these advantages, stem cell systems are highly complex and pose unique analytical challenges. Cultures often contain heterogenous cell types, from undifferentiated self-renewing ESCs to early developmental endoderm-like cells [31]. Given this inherent complexity, machine learning methods cannot be used to produce molecular models with mechanistic details based on high-throughput data. However, they can provide an “impressionistic” view of molecular interactions and hypothesize novel protein associations for experimental validation [17], [18].

Previous mESC-specific computational studies relied on limited amounts of high-throughput input data, all of which was considered equally reliable. For example, the Integrated Stem Cell Molecular Interaction Database (iScMiD), combined data from 12 different studies (mostly ChIP-Chip) to create a consensus network of ∼50 K edges [32], [33]. Others have constructed networks to investigate aspects of self-renewal by analyzing a subset of growth conditions, perturbations, and data types [34], [35]. In contrast, we use statistical machine-learning techniques to integrate a much larger, more diverse mESC data compendium (representing work from 60 studies and 6 experimental techniques) and identify novel functional relationships among proteins. By focusing on a single stem-cell system, at a specific developmental stage, within the context of well-defined biological processes, we produce consensus predictive networks with greater biological relevance than those made using generalized, context-independent methods.

## Results

**Figure 2:**
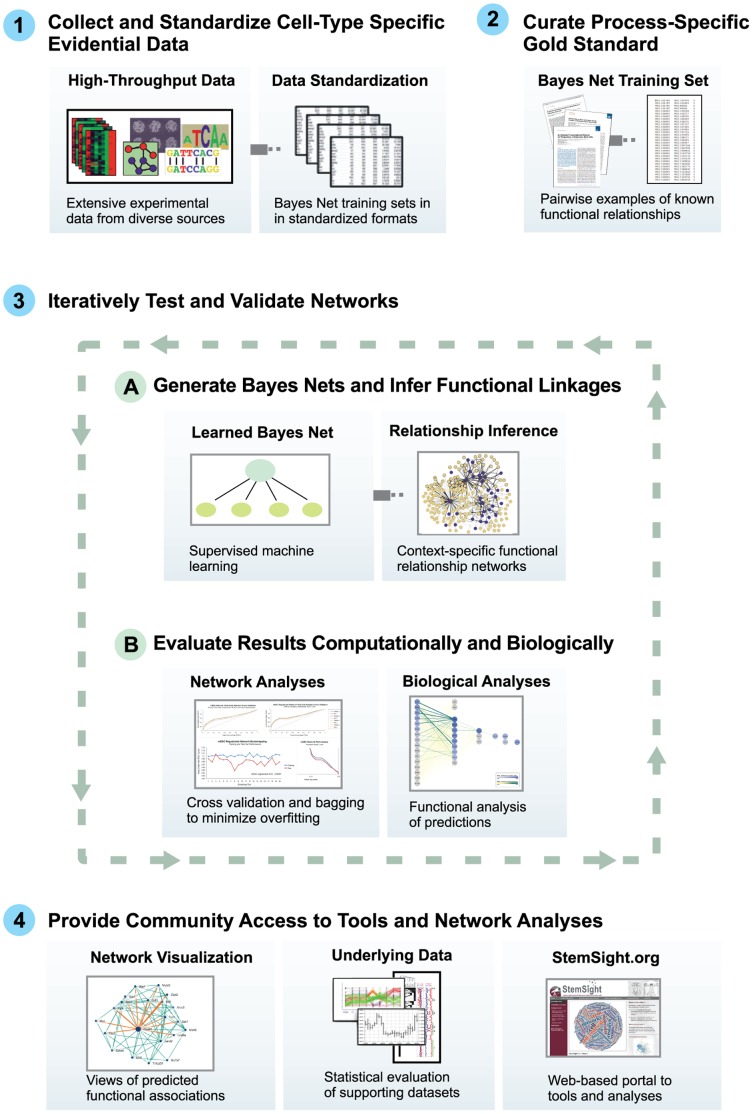
Cell-Type-Specific Data Integration and Machine-Learning Methodology. Our approach is designed to generate reliable and relevant predictive biological networks using high-throughput data limited to a specific cell type and a training gold standard focused on biological processes active in that cell type. This process can be distilled into four basic steps: **1.** Collect and standardize cell-type specific data from studies using diverse high-throughput experimental techniques, including microarray gene expression, chromatin immunoprecipitation (ChIP) on chip (ChIP-Chip), ChIP followed by high-throughput-sequencing (ChIP-Seq), affinity purification followed by mass spectrometry (AP-MS), whole-genome small interfering RNA (siRNA) screens, and phylogenetic sequence similarity. For this case study, we focused on mouse embryonic stem cell (mESC) data. **2.** Curate a process-specific gold standard training set to provide a baseline for assessing data reliability and significance for related biological processes known to be active in the cell type of interest. Our gold standard training set consists of experimentally validated pair-wise associations between genes and proteins known to be involved in mESC self-renewal, pluripotency, and cell fate determination. **3.** Iteratively test and validate networks. *A.* Use a naïve Bayesian network classifier to perform inference and predict novel gene and protein relationships. Our network predicts pairwise functional associations that influence mESC self-renewal and early developmental processes. *B.* Validate the accuracy of predicted functional relationships using standard machine learning performance metrics, cross validation, and bootstrapping, followed by evaluation of biological content. Our protocol for assessing networks ensures our results are highly reliable and relevant to mESC self-renewal. **4.** Provide community access to analyses and tools. Through StemSight.org, we provide access to network analyses and visualization tools to enable users to further explore networks centered on their genes of interest.

We used a naïve Bayesian network methodology (Figure 2) to create a cell-type-specific predictive biological network of protein-coding genes in the context of self-renewal and closely related processes (*e.g.* pluripotency and cell fate determination) in mESCs. For our training set, we manually curated a positive reference of 2056 pair-wise gene relationships (with a prior of 1) among 354 genes associated with mESC self-renewal or annotated to signaling pathways involved in early embryonic development (Table S1), based on information extracted from 98 recent journal articles (Table S2). We automatically generated a negative reference of 20,560 protein gene pairs (with a prior of 0) not documented to be associated mESC self-renewal. We joined these references together to produce a mESC self-renewal gold standard with a class distribution of 1∶10 (positive:negative) that was used to train the Bayes net. For evidential data, we assembled a compendium of high-throughput mESC data, representing 60 independent research studies, including all mouse data used in prior mESC-focused computational efforts (Table 1; Table S3). This mESC data compendium consisted of ∼2.2 million data points, collected under 992 conditions, using 6 different high-throughput experimental techniques, and encompassing more than 6 billion gene-pair measurements. We used the trained Bayes net to make posterior predictions of functional relationships among 21,291 protein-coding mouse genes based on patterns observed in the integrated evidential data.

**Table 1:** Summary of Integrated mESC Genomic Data.

Data Type	Datasets (Platforms)	Conditions	Gene Pairs	% Supporting Top Edges*	Mean Redundancy
Gene Expression	58 (19)	807	4,843,618,683	45% | 41%	0.061028
Protein-DNA Interactions	16 (10)	183	914,929,016	55% | 58%	0.022003
Physical Interactions	1 (1)	1	207	0% | 0%	0.000004
Phylogenetic Profiles	1 (1)	1	123,284,253	0% | 0%	0.147362
Whole-Genome RNAi Screens	1(1)	2	131,795,730	0% | 0%	0.171811

* Top Ranked Edges | Top 0.01% of Edges.

**Figure 3:**
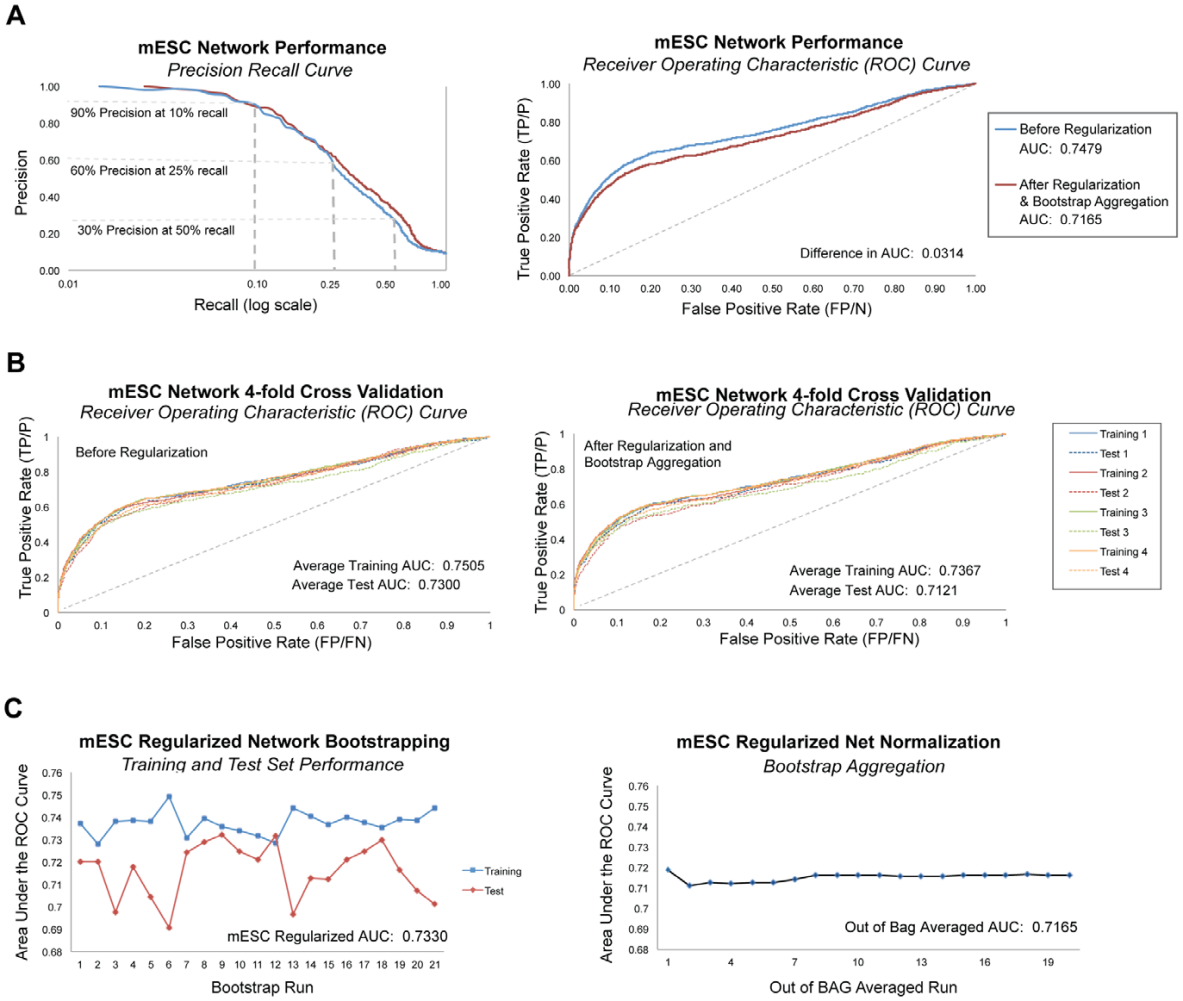
Network Performance Evaluations. **A.** Computational assessment of network performance using standard machine learning metrics showed that precision at 10% recall was 90%, and 60% at 25% recall, before and after regularization and out of bag averaging to correct for overfitting to noise. The area under the Receiver Operating Characteristic (ROC) curve (AUC) for the mESC network was 0.7479; after regularization and out of bag averaging, the AUC was 0.7165. **B.** We conducted 4-fold network cross validations by removing 25% of edges in the gold standard (4-fold Gold Standard). ROC curves showed a small amount of overfitting, most apparent in cross validations for which we removed 25% of genes (rather than edges) from the network training set (Figure S1). **C.** We conducted 20 bootstrap runs, using 70–30 split (training to test) of the gold standard answer file, and performed out-of-bag averaging to produce a single network. The relatively flat trend of AUC over out-of-bag-averaging runs confirms the minimal amount of overfitting and produced a single network with high confidence inference scores.

*Notes:* A total of 77 high-throughput datasets were collected from various public sources to create a compendium of mESC-specific data that included 992 conditions (e.g. columns in a microarray matrix) and ∼2.2 million data points (Table S3). These data were standardized and integrated into ∼6 billion gene/protein pairs, and used as evidential data to generate a predictive mESC-specific network focused on mESC self-renewal and cell fate. Datasets were weighted based on the amount of shared mutual information contained in each as compared to all evidential datasets used by the Bayes net. A low mean redundancy indicates the dataset is highly unique. As observed in other similar Bayesian network data integration efforts (including integration of human data), genetic and physical interaction data were the most reliable, but also the least common [11]. We strove to assemble a diverse and comprehensive set of mESC data that would provide the most coverage and be highly informative. Protein-DNA Interaction data included chromatin immunoprecipitation (ChIP) followed by microarray hybridization (ChIP-Chip) and ChIP followed by high-throughput RNA sequencing (ChIP-Seq). Top ranked edges were the 639 edges with a rank order of 1 and an inferred edge weight ≥0.9999 (Figure 3A, Table S11); the top 0.01% of the network consists of the 22,664 edges with an inferred edge weight ≥0.9966 (Figure 3B, dataset contributions to top 0.01% edges available at StemSight.org/stemdata.html).

### mESC-Specific Network Predicts Novel Self-renewal Proteins

The resulting undirected, predictive mESC network of ∼226 million gene pairs had 582,789 high-confidence edges with a posterior inference score of 0.9 or higher involving 8980 genes that were predicted to be strongly associated with self-renewal and cell fate in the context of mESCs. We identified 56 potential hub genes with a scaled degree of 0.55 or higher, 59% of which were novel players not included the positive gold standard. Computational evaluations showed the network achieved 90 percent precision at 10 percent recall, and had an Area Under the Receiver Operator Characteristic (ROC) curve (AUC) of 0.75 (μ = 0.7402, SD = 0.01317), which is significantly better than random (p-value = 2.685E–10) and is competitive with prior mammalian Bayes net efforts (Figure 3A) [17], [19]. Standard machine learning metrics and cross validation revealed some evidence of overfitting (*i.e.* tailoring a solution so tightly to the training data that the Bayes net does not learn to generalize the trend and recognize new examples) (Figure 3B). Regularization and bootstrap aggregation minimized overfitting at the cost of reducing the AUC to 0.72 (Figure 3C, Figure S1A) [7], [10], [19], [36], [37]. Top ranked, high confidence edges in this network were supported by a diversity of high-throughput data, but predominantly by Protein-DNA binding data similarity profiles (Table 1, Figure S3).

To assess biological content and functional relevance of our mESC network, we used functional genomics tools [28], [38] to evaluate GO term enrichment, validate that gene pairs known to significantly influence mESC self-renewal were strongly connected in the probabilistic network, and identify novel genes with strong functional linkages supported by evidential data. We used standard methods for weighted network analysis to investigate network topology, identify major hubs, and search for novel interactors. Our results confirm the roles of genes and proteins known to be involved in early developmental transcriptional regulation and stem cell maintenance, including *Pou5f1* (also known as *Oct4*), *Sox2, Nanog, Klf4, Suz12, Phc1,* and *Trim28,* all of which were major hubs in our mESC-specific network. Functional annotation analysis showed the most strongly connected genes in the mESC network were highly enriched for stem-cell-related biological processes, including development, maintenance, and differentiation, as well as transcriptional regulation (Table S4).

To identify the tightly connected “core” of our gold standard training set, we evaluated the distribution of predicted posterior edge values and identified a edge cutoff of 0.25 (Figure S2A). We then calculated a functional correlation score to the 356 genes involved in positive gold standard edges to identify top self-renewal gene hubs. We consider any training set gene with at least one strong connection (≥0.25) to another member of the training set as a more reliable member of the gold standard: a “golden” gold standard gene. We used a “guilt by association” metric to measure the strength of functional linkage between a given gene and genes in our “golden” gene set. We refer to this measure as the Self-Renewal Correlation score (SRC; details in *Materials and Methods*), which we use to evaluate the likelihood of novel gene association with self-renewal programs and to reassess the role of genes included the gold standard (Figure S2C). For example, many genes one would expect to have high SRCs are key self-renewal players such as *Pou5f1* (SRC: 1.0000), *Sox2* (SRC: 0.9505), and *Gdf3* (SRC: 0.9419), while others exhibited low SRCs, such as *Pdc* (SRC: 0.0104) and *Smad9* (SRC: 0.0451), and are thus less likely to be involved in self-renewal and closely related early developmental processes.

In addition to the known gold standard genes, we found many novel genes exhibited high correlation to self-renewal proteins based on network connectivity and SRC (Table 2). These genes included: Gbx2, *Jarid2, Tcea3, Tdgf1, Msh6, Slc3a2, Ifitm1, Tdh, Reep3, Jam2, Rpp25, Trh, Msx2, Zfp428, Tfcp2l1, Etv5*. This list is enriched for genes known to play a role in cell fate determination and other early developmental processes as well as genes involved in transcriptional regulation and DNA binding. For example, *Jarid2* (SRC: 0.9235) is a regulatory subunit of Polycomb Repressive Complex 2 (PRC2), which is involved in repression of genes important for development and cell fate specification [39]. *Gbx2 (SRC: 0.9367)*, a transcription factor linked to stem cell pluripotency and differentiation in developing embryos, is a direct target gene of WNT signaling known to be involved in neural crest induction as well as specification and formation of the neuroectoderm [40], [41]. However, none of these genes were included in our training set, demonstrating the ability of the Bayes net to predict potentially meaningful novel players in this biological context.

**Table 2:** Candidate Genes for Experimental Validation.

Novel Gene	SRC (Scaled)	Novel Gene	K (Scaled)
Gbx2	0.9367	Gbx2	0.6259
Jarid2	0.9235	**Tcea3**	**0.6399**
**Tcea3**	**0.9234**	**Msh6**	**0.6292**
Tdgf1	0.9013	Zfp296	0.6067
**Msh6**	**0.9080**	Socs2	0. 5583
Slc3a2	0.9085	Slc3a2	0.6124
Ifitm1	0.9092	**Tdh**	**0.6087**
**Tdh**	**0.9056**	Rpp25	0.5973
**Reep3**	**0.8971**	Tdgf1	0.5184
**Jam2**	**0.8945**	Etv5	0.5844
Rpp25	0.9047	Zfp428	0.5927
Trh	0.8877	Rhob	0.5820
Msx2	0.8932	**Reep3**	**0.5844**
Akap12	0.8865	Akap12	0.5771
Cited2	0.8946	Ifitm1	0.5816
Etv5	0.8855	**Jam2**	**0.5798**
Crmp1	0.8754	Mcl1	0.5731
Mcl1	0.8843	Nolc1	0.5729
Rhob	0.8964	Abcc4	0.5739
Gjb3	0.8907	Dnmt3l	0.5693
Zfp428	0.8858	Plcg2	0.5671
Mkrn1	0.8752	Cited2	0.5735
Zfp296	0.8590	Upp1	0.5571
Anp32a	0.8651	Jarid2	0.5657
Upp1	0.8589	Anp32a	0.5722
Dbf4	0.8777	Dbf4	0.5708
H2afx	0.8526	Trh	0.5689
Zswim1	0.8713	Lpp	0.5507
Slc7a7	0.8503	Ina	0.5499
Slc7a3	0.8675	Tcfcp2l1	0. 4887

*Notes:* We ranked genes not included in our mESC gold standard by network topology measures: self-renewal correlation (SRC) and scaled network degree (K). We used network degree to identify hubs and topologically important gene nodes, and SRC scores to discover genes functionally related to mESC self-renewal. Our highest confidence potentially novel self-renewal genes (in bold) ranked high in both gene lists and were not yet annotated to biological processes associated with self-renewal or cell fate determination.

**Figure 4:**
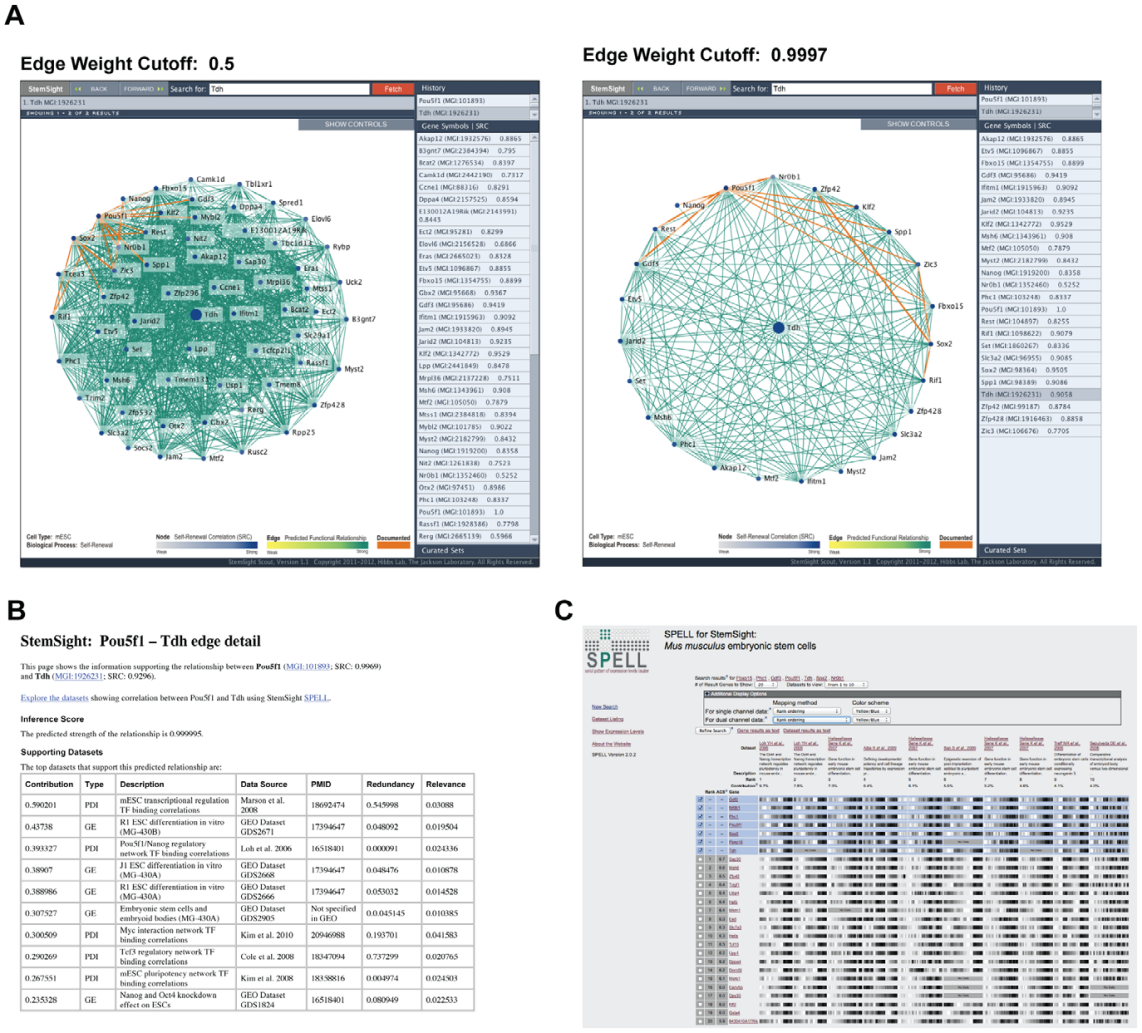
Data Visualization for Mining mESC Self-Renewal Gene Predictions. **A.** Views of *Tdh-*centric networks created using our StemSight Scout visualization tool, available at StemSight.org. Adjusting Scout network views to display only edges with inference scores of 0.5 and 0.9997 show that the novel gene *Tdh* is tightly connected to many well-known self-renewal genes in our training gold standard, including Pou5f1, Sox2, Nanog, Nr0B1, and Phc1. **B.** Supporting edge info for the *Tdh* – *Pou5f1* edge. Supporting edge information shows that this edge is supported by several protein-DNA interaction (PDI) assays as well as gene expression datasets from a study investigating mESC cell differentiation in different mESC cell lines. For supporting edge detail between *Tdh* and other gold standard genes, see Figure S5 or explore the *Tdh* interactome online at StemSight.org/scout. **C.** SPELL for StemSight Search Results. From a supporting edge information window, you can drill down to the individual gene expression levels in microarray datasets. This view shows how expression data reveals rank-ordered correlations observed between *Tdh* and gold standard genes *Gbf3, Fbxo15, Nr0b1, Phc1, Pou5f1,* and *Sox2.*

L-threonine dehydrogenase (*Tdh; SRC: 0.9058*) is one of the less well-studied genes in our list of high-confidence novel gene candidates for experimental validation that was strongly predicted to be involved in self-renewal, pluripotency and cell fate, and tightly linked to many of our “golden” gold standard genes, including *Pou5f1, Sox2, Nanog, Nr0b1, and Rif1,* (Figure 4A, Figure S5). *Tdh* catabolizes threonine into glycine and acetyl-CoA, which is used by the TCA cycle to generate ATP. While there were no GO annotations for this gene based on experimental data at the time we developed our training set, nor articles about the role of Tdh in mESCs at the time we created our gold standard, recently published articles confirmed that mESCs are dependent on threonine catabolism to support accelerated cell cycle kinetics [42], [43]. To learn more about the underlying datasets that support functional linkages between *Tdh* and key self-renewal genes, such as *Pou5f1*, we evaluated Bayes net statistics for edge weight and top supporting datasets (Figure 4B,C). These statistics showed that the functional relationship between *Tdh* and *Pou5f1* was supported by ChIP-Chip binding data from five different studies investigating the regulatory circuitry of mESCs and microarray data from a study analyzing mESC differentiation. Tdh connections to other golden gold standard genes were largely supported by the same type of ChIP-Chip data (Figure S5). By drilling down to the most reliable datasets, as determined by our machine learning evaluations, we were able to quickly identify *Tdh* as a potential target of the core regulatory circuitry of mESC self-renewal and pluripotency [3], [44] to manage cell-cycle controls during the rapid growth phase of early embryonic development.

We used the SRC metric to select our top 10 candidate genes for experimental validation: Tcea3 (SRC: 0.9234), Msh6 (SRC: 0.9080), Reep3 (SRC: 0.8971), Jam2 (SRC: 0.8945), Crmp1 (SRC: 0.8754), H2afx (SRC:0.8526), Nolc1 (SRC: 0.8476), Klf9 (SRC: 0.8616), Creb3 (SRC: 0.8447), and Myst2 (SRC: 0.8432). This list of novel genes predicted to be associated with mESC self-renewal includes several transcription factors and chromatin modifiers; all have high SRCs, but no GO annotations related to early embryonic development processes.

### Appropriate Evidential Data is Critical for Useful Network Predictions

To further assess the impact of cell-type-specific evidential data on Bayes net predictions, we prepared three additional input data feature sets by varying the amount, diversity, and appropriateness of evidential mouse data. We generated test networks for each of these feature sets, using the same mESC self-renewal training set and evaluation metrics as for our mESC-specific network.

To evaluate network performance using a relatively small amount of inappropriate data (not specific to mESCs), we trained a Bayes net using a minimalist library of 16 datasets, representing ∼300 experimental conditions (Table S5). This feature set, composed primarily of non-cell-type-specific data downloaded from molecular interaction databases, was similar to evidential data compendiums used for prior functional relationship network projects using mouse data [17], [18] and a mouse gene function prediction competition [20]. Networks generated using this minimalist input data and our self-renewal training set produced the lowest AUC (0.5931) and exhibited the least evidence of overfitting. There was insufficient data to perform regularization, which is best applied to very large-scale data integration [19]. The resulting network contained no notable network hubs and had only 543 edges (involving 446 genes) with an inferred probability of a functional relationship greater than 0.9. Of these edges, only one involved canonical embryonic stem cell self-renewal factors (*Pou5f1– Nanog*; weight = 0.9754). The remaining edges were a random assortment of loose connections between genes annotated to disparate functions. These functionally vague results confirmed that limited evidential data, while potentially useful for more general gene function studies [17], [18], are inappropriate for exploring a context-specific cellular process, such as mESC self-renewal.

**Figure 5:**
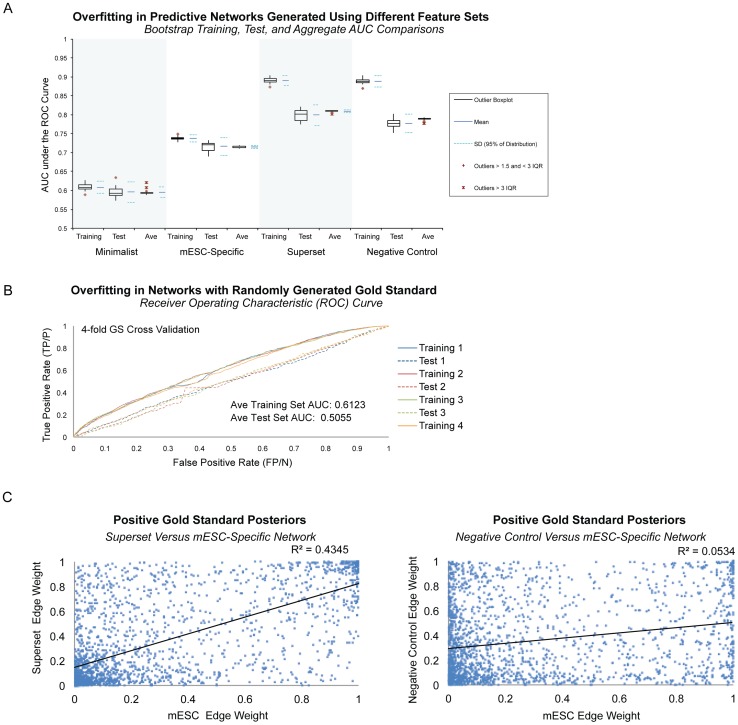
Importance of Feature Selection in Bayesian Network Machine Learning. A. Networks trained using the same mESC gold standard but different feature sets had markedly different evidence of overfitting. We generated networks using three different feature sets: a minimalist library of 16 datasets composed largely of non-cell-type-specific data from molecular interaction databases, our mESC-specific compendium composed of 164 datasets restricted to mouse mESC data and a small amount of data not specific to any cell type, a superset compendium composed of all mESC training data plus an additional 646 non-tissue specific mouse microarrays, and a negative control compendium containing all datasets except those with mESC data. Using machine learning metrics, we found that the network trained on a small amount of non-tissue-specific data achieved the lowest ROC curve AUCs and had the least amount of overfitting. The mESC-specific network achieved a higher AUC, and showed evidence of minimal overfitting. The superset and negative control networks had the highest AUCs, but also showed extreme overfitting with a difference of greater than 0.1 between training and test set AUCs. Bootstrapping followed by out of bag averaging largely correct for overfitting in the mESC-specific, superset, and negative control networks. However network content varied dramatically. **B.** Overfitting in Networks with Randomly Generated Gold Standards. Networks trained on randomly generated gold standards performed better than random according to standard machine learning metrics, but 4-fold cross validation revealed these networks had evidence of overfitting that could be corrected for using regularization and bagging techniques. **C.** Evaluating Network Differences using Positive Gold Standard Posteriors. A scatterplot of superset versus mESC-only network positive gold standard posterior edge (those with a prior of 1) illustrates that while there is relatively high correlation (Pearson correlation r = 0.6592), there is also a broad range of disparity between the two networks. A scatterplot of negative control versus mESC shows that there is less correlation between the two networks (Pearson correlation r = 0.2311), and reveals the subset of the training gold standard supported by non-mESC data.

As a negative control, we assembled a feature set composed of a large amount of inappropriate data: 656 datasets from a broad range of mouse tissues and cell types, *excluding* mESCs (Table S6). This feature set was composed largely of microarray data and spanned ∼13,500 experimental conditions. To further explore the impact of using a combination of *any* type of mouse data, we created a feature “superset” based on a sprawling compendium of all available high-throughput mouse data, including data from our negative control, minimalist set, and mESC-specific datasets: a total of 810 datasets representing ∼14,500 conditions (Table S7). Both the negative control and superset networks achieved higher AUCs than the mESC network (0.88 and 0.86, respectively), but they also exhibited more dramatic evidence of overfitting (Figure 5A). This was not unexpected as the number of features in these test sets far exceeded the number of genes in the training set. Subsequently, the Bayes net was able to find patterns in the noise of the input data that most likely did not reflect real biology, often manifested as over-inflated results. Overfitting in networks generated using the superset of input data was even more apparent when trained on randomly generated, negative control gold standards. These test networks all achieved AUCs in the mid-to-high 0.80 s; however, overfitting was largely mitigated by regularization and bootstrap aggregation, which reduced test AUCs back to the expected random levels (∼0.5) (Figure 5B).

To evaluate the impact of data compendium size as well as composition, we conducted a series of data compendium tests using the type of gene expression and molecular interaction data included as evidence in all three test networks (Figure S4). AUCs for networks generated using incrementally increasing numbers of randomly selected mESC gene expression data sets plateaued at ∼0.65 as the size of the compendium reached ∼45 datasets (∼600–700 conditions). Test networks generated using the same mESC gold standard and different compendiums composed of 60 non-cell type specific mouse datasets achieved slightly higher AUCs than a mESC test network based on 58 mESC specific gene expression datasets, and showed evidence of roughly the same amount of overfitting. However, biologically, these networks were all quite different. Only the mESC gene expression test network had high-confidence edges with a posterior edge weight of 1 involving genes highly enriched for biological processes associated with stem cell self-renewal and embryonic development. Even so, this mESC gene-expression-based network was not as biologically relevant or reliable as networks generated using more diverse mESC evidential data (Figure S4, Table S8).

### Computational Performance Metrics do not Necessarily Measure Biological Relevance

When evaluated using traditional machine learning metrics, the negative control and superset network computationally performed better than the mESC-only network. Even after regularization and bootstrap aggregation, both of these test networks achieved AUCs of ∼0.80, as compared to 0.72 for our mESC-specific network. However, they were very different networks, capturing very different flavors of biological information (Table S9). Top network hubs in the negative control network were enriched for biological processes associated with FGFR signaling, MAPK signaling, regulation of cell proliferation, gene expression, and transcription. In contrast, the superset network (which included negative control data as well as mESC-specific data) was enriched for many of the same documented self-renewal functional associations found in the mESC-only network, but the signal was “blurred” in comparison. For example, out of a total of 21,291 protein-coding genes, *Pou5f1, Nanog,* Sox2, and *Suz12* emerged as the most highly connected network hubs in the superset network, after which there was a steep drop-off in degree (Table 3). These four genes were also the top hubs in our mESC network, but they were even more tightly connected, with a higher mean degree. In fact, while much of the gold standard was similarly supported by both the mESC-specific and superset data collections, (Figure 5C; Pearson’s correlation r = 0.6592, r^2^ = 0.4345), there was a broad range of functional disparity between the two networks. Not surprisingly, this difference in functional linkage was even more evident when comparing the negative control to mESC posterior gold standard edges. (Figure 5C; Pearson’s correlation r = 0.2311, r^2^ = 0.0534). In general, high-confidence gold standard genes in the negative control and superset networks were involved in signaling pathways known to be active in both adult and embryonic tissues (Table S10). The points of agreement between networks indicate that either 1) some genes are so strongly connected in the context of mESC self-renewal that additional non-mESC-specific datasets in the superset did not obscure the signal or 2) these genes are strongly connected in multiple cellular and process contexts. Thus, the additional inappropriate datasets used as evidence in the test networks tended to include information not specifically related to stem cell self-renewal in mESCs, resulting in less focused, less biologically meaningful networks, despite seemingly improved computational performance metrics.

**Table 3:** Comparison mESC-Specific and Test Network Connectivity.

Mean Degree	mESC-Specific	Mmu Superset	Negative Control	Minimalist
≥ 0.30	0.056%	0.018%	0.005%	0%
0.20–0.30	2.404%	0.032%	0.655%	0%
0.10–0.20	22.12%	3.35%	18.12%	0%
0.00–0.10	75.42%	96.60%	81.22%	100%

*Notes:* The percentage of strongly connected gene hubs (those with a mean degree greater than 0.2 out of a total of 21,291 protein coding genes) is markedly higher in the mESC-specific network as compared to the superset or negative control networks. Degree is a measure that reflects the number of genes within the network that are predicted to be functionally linked to a given gene. In these networks, which were trained using a gold standard focused on mESC self-renewal, a higher mean degree indicated that the given gene is more likely to interact with multiple other genes, and tended to be enriched for mESC-specific self-renewal processes. Highly connected genes in the negative control network were predominantly annotated to biological processes related to self-renewal functions that are active in all cell types, such as transcriptional regulation, cell proliferation, as well as developmental processes associated with multiple cell types, such as embryonic morphogenesis.

### Network Visualization Reveals Novel Functional Relationships

To make our predictive mESC network readily available to the stem cell research community, we created an interactive, online visualization resource at StemSight.org. A flat file of the predictive mESC network, containing all edges with an inferred probability of functional relationship ≥ 0.2 (18,097,736 edges) is available for download at this site. For those uncomfortable working with large graph files, this network may be explored online using StemSight Scout (StemSight.org/scout). The Scout dynamic visualization interface, implemented using ThinkMap visualization technology, highlights potentially novel self-renewal genes by coloring nodes based on their SRC score and illustrates the weight of predicted interactions by coloring edges based on the inferred posterior probabilities. Documented self-renewal genes and edges, those included in our gold standard training set or other curated sets, are also color-coded, making it easy to visually segregate novel from known. If a displayed edge is in the positive gold standard training set, links are provided to the original articles documenting the relationship. With Scout, users can search for and download information about interactomes centered around a gene of interest, view predicted subnetworks for sets of self-renewal genes identified by previous computational studies, and “drill down” into the data underlying predictions (Figure S4, Table S11). Visualization of underlying gene expression levels is provided through a mESC-specific instance of the Serial Patterns of Expression Level Locator (SPELL) system [45], which reveals gene expression correlations from the mESC microarray datasets used to train the classifier (Table S3).

Through the resources available at StemSight.org, we facilitate analyses for which a greater understanding of the underlying data can be informative, and effectively extend the shelf-life, accessibility, and usefulness of existing high-throughput stem cell data in the literature. Furthermore, by focusing user attention on the most reliable datasets for their area of biology, as determined by our machine learning evaluations, we provide a framework for gene function discovery in the context of mESC self-renewal.

## Discussion

### Statistical Analysis of Input Data Enhances Relevance of Biological Networks

**Figure 6:**
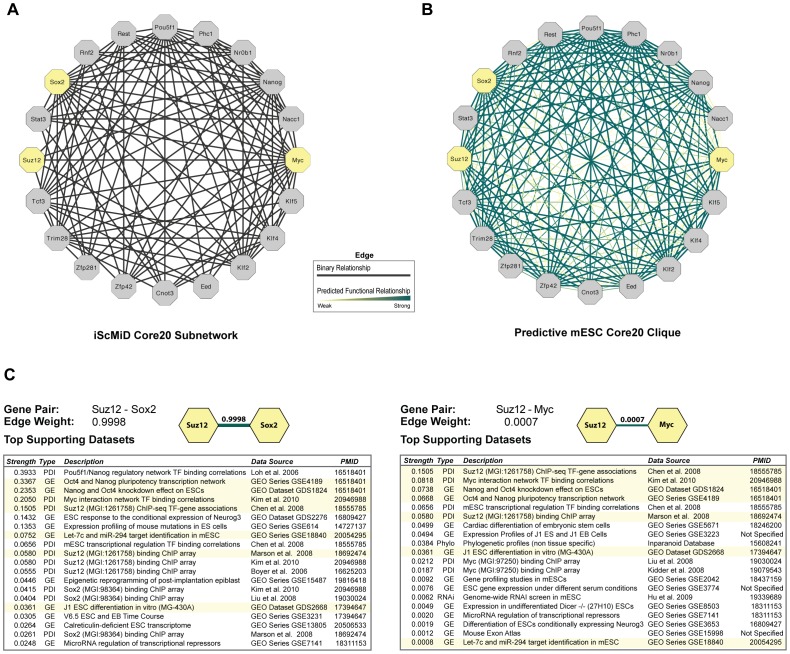
Advantages of Statistically Principled Approach. A. The iScMiD Core20 subnetwork of transcription factors used as bait in the 12 studies included in the iScMiD integrated mESC database [34], [35], recreated as an undirected graph using edges available from the iScMiD website. In the iScMiD network, all edges have equal weight and all high-throughput data is considered equally reliable, hence the authors note there may be many false positives. **B.** The fully connected clique of mESC network posteriors for the iScMiD Core20 transcription factors predicts connections not shown in iScMiD and reveals potential false positives as not all connections are equally supported by the evidential data. For comparison, we checked underlying data for two edges, highlighted in yellow, one of which is not supported in the iScMiD subnetwork (Suz12– Sox2), and one which is only weakly supported in our mESC-only network (Suz12– Myc). **C.** Contrasting detailed information about underlying data supporting the strong functional linkage between *Suz12*– *Sox2* (Edge Weight: 0.9998) versus the weak linkage between *Suz12*– *Myc* (Edge Weight: 0.0007) shows that top supporting datasets vary from edge to edge and that the strength of dataset contribution to edge weight may differ significantly. (Highlighted rows are datasets that support both edges.).

**Figure 7:**
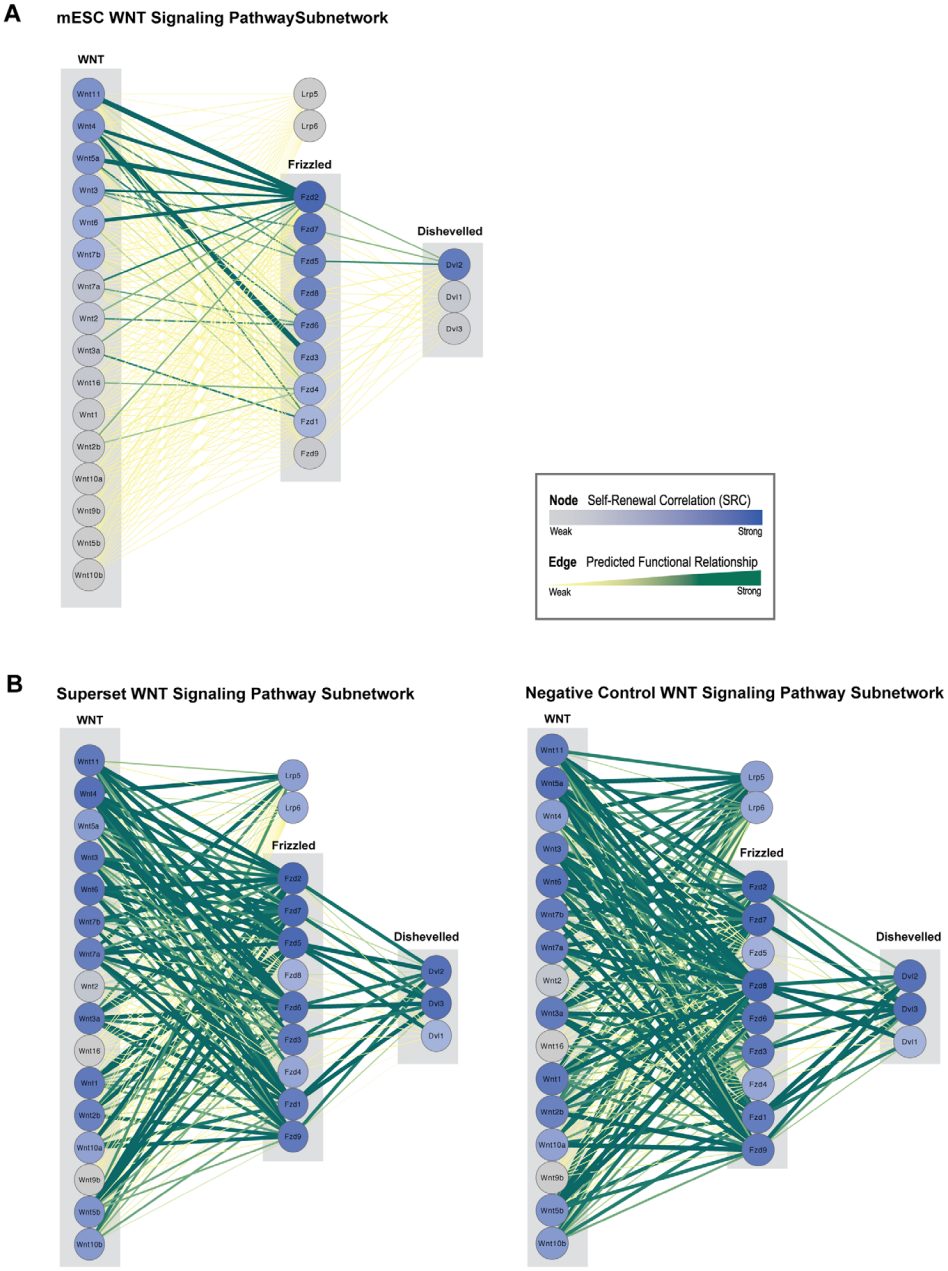
Comparing Subnetworks of WNT Signaling Pathway Participants. A. WNT Signaling Pathway Subnetwork. A model of the WNT Signaling pathway adapted from the curated KEGG pathway for *M. musculus* (Mmu) includes SRCs for Wnt, Frizzled, and Dishevelled pathway participants, illustrating that not all family members are equally supported by evidential data. Curated pathways, which are cell-type agnostic, cannot capture these differences in connectivity. A corresponding network of mESC posterior edges involved in this view of WNT Signaling (created in Cytoscape) demonstrates the variance in edge weights and SRCs in the signaling cascade. **B.** The same WNT Signaling subnetwork produced using Mmu superset and negative control posterior edge weights and SRCs captures a different picture of connectivity as compared to the mESC network. Far more WNT signaling activity between different WNT family member ligands and Frizzled receptors is evident in the test subnetworks. This may reflect WNT signaling activity observed in data from both mESCs and other cellular contexts in the Mmu superset of features. The influence of WNT signaling in other cellular contexts is even stronger in the negative control subnetwork.

The supervised machine learning approach we used minimizes bias by statistically evaluating data relevance, including which experimental designs are most appropriate and which conditions are most informative. Bayes nets assigned a statistical level of confidence for each input dataset; regularization filtered redundant mutual information shared among datasets. Our method enabled us to report both the strength of the relationship between gene pairs (edge weight) and statistics that describe which evidential data contributed to each edge (Figure 6A, B). Using this information, we could “cross validate” predictions made in other studies, such as functional linkages between transcription factors that have been computationally validated as essential for mESC pluripotency [33], [34] (Figure 7A). For example, our results predict a strong functional linkage between *Suz12– Sox2* (edge weight: 0.9998), but a weak connection between *Suz12– Myc* (edge weight: 0.0007). Closer inspection of these edges reveals that although more than half of the top supporting datasets are the same for each edge, the contribution strength of evidence often differs significantly (Figure 6C) because our approach includes degrees of co-expression, binding affinity, *etc.,* that are not considered when using a binary network construction approach. In this way, our weighted network provides a view that is closer to biological reality as few genes function in a binary fashion in *any* system context.

### Tailoring Prior Knowledge to a Single Cell-type Clarifies Results

For our mESC gold standard, we intentionally included interactions that influence closely related developmental processes, especially when cell fate hinges on stoichiometry or epigenetic regulation of a common set of genes. For example, normal expression levels of the *Pou5f1* transcription factor support self-renewal, while aberrant over expression (as little as two-fold increase) can induce premature differentiation into primitive endoderm and mesoderm cells, and loss of *Pou5f1* induces differentiation to trophectoderm cells [46], [47]. For developmental signaling pathways, we included direct and indirect connections between all pathway participants, unless cell-type-specific players were indicated in the literature. For example, for the WNT signaling pathway, we included all 16 Wnt ligands, all 9 Frizzled receptors and all 3 Dishevelled signal transducers in our mESC gold standard. For JAK/STAT signaling, the literature was more explicit with respect to ligand-receptor pairs; subsequently, our gold standard includes only references to the IL6 class of cytokines and cytokine receptors that are important for mESC biology (LIF, LIFR, and IL6ST) [48], [49].

Despite our extensive manual curation efforts, the examples in our training set are of variable quality and reliability. Some interactions, such as *Pou5f1– Nanog* were referenced and observed multiple times, while others such as *Yy1*– *Cbx2,* were included as training examples based on less well established experimental evidence. Subsequently, some of our training edges may ultimately prove unreliable. This is especially true for examples derived from signaling pathways (such as WNT), where we included all possible ligand-receptor pairs in the absence of other information. Based on our results, only a subset of these possible interactions appears to be important for mESC biology.

Our SRC measure may prove a useful tool for assessing which gene family members are more likely to be active in a signaling pathway within the context of a specific cell type or developmental stage. To demonstrate this application, we extracted gold standard edges involved in the WNT signaling pathway (adapted from KEGG), and ranked possible Wnt, Frizzled, and Dishevelled participants by SRC (Figure 7A). Our mESC WNT pathway highlights which components are strongly supported by mESC data and may help identify interactors required to activate canonical and non-canonical WNT signaling cascades that influence mESC self-renewal, pluripotency, and cell fate [50]–[52]. For example, we observed high SRCs for *Wnt11, Wnt5a, Wnt4,* and *Fzd5*, which have been shown to work together in a context-dependent manner to activate canonical WNT signaling [53]. In addition, our network captures relationships among WNT family members known to work in concert to mediate signaling activity. *Wnt5a* has been shown to compete with *Wnt3a* for *Fzd2* receptor binding sites [50], and this *Wnt5a – Wnt3a – Fzd2* triad is strongly supported by our posterior weights.

Comparison of our mESC-specific-network to the negative control and superset networks further illustrates how predicted functional linkages may be used differentially to identify specific ligand-receptor pairs active in mESCs signaling pathways. While our mESC-specific network predicts only a few specific edges between pathway participants (Figure 7A), in our negative control and superset networks, all 16 Wnt ligands are almost equally correlated with known self-renewal genes, and all but 3 Wnts (*Wnt2, Wnt16,* and *Wnt10b*) were strongly linked with most Frizzled receptors (with the exception of *Fzd5*) (Figure 7B, Table S10). These subnetwork views show that in the superset and negative control networks there was evidence of general WNT signaling activity linking most WNT ligands and receptors in some cellular context. Because all WNT ligand-receptor pairs were documented in our training set, the superset and negative control networks were better able to capture all of WNT signaling in mouse, whereas our mESC network results are more specific to mESC self-renewal.

Our results support the importance of cell-type-specific data integration and manually curated gold standards for Bayes net machine learning techniques, and illustrate how networks can be used to create biological-process focused predictive networks. With a better understanding of the tradeoffs involved with gold standard composition, particularly in terms of traditional machine learning metrics [10], [54], one can develop different, yet complementary training sets to explore different facets of biological relationships in the context of a given cell type. This approach may prove particularly useful for predicting functional linkages among families of genes involved in signaling pathways active in many cells and during multiple developmental stages, and for which experimentally validated knowledge of the role of specific pathway participants is sparse.

### Cellular Context Matters in Predictive Biological Networks

Our mESC network is clearly enriched for self-renewal and early developmental processes, and the top, most highly connected hubs (*Pou5f1*, Nanog, Sox2, and Suz12) are genes experimentally validated to influence mESC self-renewal. In contrast, other efforts utilizing more generalized GO-based gold standards for gene function prediction capture a completely different connectivity picture, where the major hubs are *Brca1, Trp53,* and *Rb1*, and the core self-renewal transcriptional regulator *Pou5f1* is connected only to *Nanog* [18]. This is not to say that general co-annotation training sets are not informative, but rather to emphasize that in complex biological systems, context matters. These approaches have demonstrated value for gene function prediction, but they may not be the best choice for exploring more specific functional associations within a defined cellular context.

The disparity between computational performance and network relevance observed in our test networks is most likely because of the composition of our gold standard training set, which included not only direct edges experimentally validated to be involved in mESC self-renewal, but also more generic direct and indirect edges associated with signaling pathways (such as WNT) active in many tissue types, not just mESCs (Figure 7B). These more general relationships were strongly supported by the non-mESC data included as evidence in our negative control and superset test networks, and subsequently credited as correct results in our computational performance evaluations. Conversely, the absence of evidential support for these generic edges in the mESC network (Figure 7A) resulted in a lower computational performance score, even though the mESC-specific network provided a more reliable depiction of gene functional relationships within the context mESC biology. The differences among our mESC-specific and test networks demonstrate the tradeoff in using computational performance evaluations that are blind to cell-type-specificity and the challenge of gold standard development for context-specific network prediction, especially when the goal is to not only recapitulate what is known, but also to discover novel biology at the cellular level.

### Conclusions

We have shown that naïve Bayesian networks trained using a biological-process-specific gold standard and cell-type-specific evidential data can provide useful, testable insights into novel biology. Our results underscore that traditional machine learning performance metrics alone are not sufficient for evaluation of the predictive accuracy of complex biological networks, particularly when the goal is discovery of novel gene/protein interactions within a defined cellular context. High AUCs may reflect how well a network recapitulates what is *known*, but they are not the best measure of *unknown* biology, which doesn’t always play by predictable rules [9], [55], [56]. Reassessments of functional relationship network predictions have observed that study biases, annotation biases, data correlation structures, and high levels of noise can easily mislead machine learning approaches and, consequently, impair biological interpretation of results [55], [57], [58]. In this work, we address many of these potential pitfalls. By manually curating our training sets, we avoid annotation biases in resources, such as GO and KEGG. By restricting evidential data to our cell type of interest, we reduce the impact of multi-functional genes. We perform extensive network regularization to manage data correlations and biases, and do not rely solely on traditional machine learning performance metrics to assess network quality. In this way, we are able to generate predictive biological networks that more closely reflect biological reality than other, more generalized approaches can achieve.

It is vital to assess the biological relevance of predictive networks in terms of the cellular context of interest. Just as no one type of experiment can elucidate all facets of biological pathways and mechanisms, no one network, regardless of its computational performance, will excel at making predictions about gene function in all contexts. As such, biologists should be both wary of and savvy about which computational tools and databases best support their research efforts, and preferably, use a consensus approach involving multiple computational resources. Predictive networks, such as ours, can aid in preliminary analyses by providing a comprehensive view of information otherwise lost in vast repositories of high-throughput data, but they should not be the only reference tool or method used.

In this study, we suggest alternative analytical approaches that can be used to assess novel biological predictions. We demonstrte the importance of investing in manual curation, not just in terms of gold standard creation, but also for evaluating, restricting, and normalizing datasets for integration. We also highlight the limits of gold standard curation in cases where our knowledge is incomplete, and suggest strategies to identify unsupported training edges, such as those in the WNT signaling pathway, and to tease out novel interactions for potential inclusion in future training sets (as determined by SRC scores). We are currently experimentally validating top candidate genes identified through our computational and functional analyses.

Moving forward, it will be important to extend our cell-type-specific approach to additional cellular contexts. Given the complexity of self-renewal processes and the importance of cellular context, a natural extension of this work would be to evaluate, compare, and contrast the underlying molecular foundations of self-renewal in the context of different stem cell types. As high-throughput techniques and additional resources become increasingly more sophisticated and affordable, computational methods will, in turn, become even more biologically informative. Single molecule sequencing, high-throughput proteomics, flow-cytometry-sorted stem cell populations, single cell data collections, the Knockout Mouse Project [59], and the Cell Ontology [60] will all contribute to the increasing quality, breadth, and depth of consistent, developmental stage specific mammalian data. With these evolving high-throughput data, machine-learning methods such as ours will be able to produce more mechanistic predictive models that trace molecular interactions during early development and throughout a stem cell lineage.

## Materials and Methods

### Collection and Preparation of Training and Evidential Stem Cell Knowledge

Supervised Bayesian network machine learning requires a consistently integrated collection of diverse high-throughput evidential datasets, coupled with a reliable reference gold standard (prior knowledge) for training and evaluation. To ensure high quality, consistent, and comprehensive system input, we developed a rigorous protocol for gathering and preprocessing input datasets, and carefully documented our methods for developing tailored gold standards.

#### Preparation of evidential high-throughput mouse embryonic stem cell dataset compendium

We preprocessed, normalized, and standardized a comprehensive set of mESC input data from high-throughput experiments using microarrays, ChIP-Chip, ChIP-Seq, affinity purification followed by mass spectrometry (AP-MS), and whole genome small interfering RNA (siRNA) screens, plus molecular interaction and phylogenetic data not specific to any cell type. Collectively, this data represents 992 conditions and 2,258,468 data points. A complete list of data sources used is provided in Table S3. This data was mapped to MGI Gene IDs, and preprocessed into ∼6 billion pairwise values used as features for classification. Through this process, each protein-coding gene pair (in each dataset) was assigned a similarity score, based on Euclidean or Pearson correlation distance measures between genes (see Equations 1 and 4). Pearson correlations were normalized using Fisher’s Z-transform, shifted by the mean, and divided by the dataset standard deviation to yield a collection of pairwise similarity scores with an approximately normal distribution ∼N(0,1). Values were binned into discrete ranges for use as classification features in our Bayes net integration (details as follow).

***Microarray expression data.*** Raw mESC microarray expression data files were downloaded from the Gene Expression Omnibus (GEO) [61]. Microarray datasets available in Affymetrix CEL format were normalized using the Robust Multichip Average (RMA) function in Bioconductor R/affy package (version 2.5, R version 2.10.1). Brainarray ENTREZG custom chip definition files (CDFs), which reflect the most recent gene and probe sequences, were used to map probes to genes (version 12.1.0) [19], [62], [63]. To standardize microarray data downloaded from public databases, we followed this protocol: 1) Impute missing values and remove probes with few values (probes were required to be present in at least 70% of conditions to be retained) using the KNN-impute algorithm, 2) Map microarray Probe IDs to systematic MGI gene IDs, 3) Average together consistent probe values using a maximum likelihood approach, and 4) Perform numeric clean up and consolidation (as previously described) [19], [45], [64]–[66]. The resulting standardized datasets were then converted to a PreCLustered (PCL) format and distilled into a set of pairwise similarity scores using Pearson correlation followed by Fisher’s z transformation to measure the strength of the linear relationship between gene expression values for all possible gene pairs in the study (Equations 1, 2).
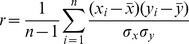
(1)

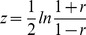
(2)Where *r* is the Pearson correlation coefficient calculated from the microarray profiles, and *z* is the Fisher Z-transformed correlation.

***Chromatin immunoprecipitation (ChIP) followed by microarray (ChIP-Chip) data.*** Raw high-throughput ChIP data was obtained from online supplemental materials and from contributing author websites. We organized data into a consistent ChIP data matrix format, mapping bait and target gene IDs to systematic MGI gene IDs. Data was processed into a pairwise format in two ways: first, we generated separate data files for each transcription factor used in the study (*e.g.* input pairs connecting each transcription factor with its putative targets as inputs or features); and second, we generated transcription-factor-binding similarity profiles between all gene pairs (*e.g.* values determined by the number of transcription factors shared by each gene pair). These similarity profiles were created by calculating dot products between vectors of individual transcription factor binding scores for each gene pair in the study (Equation 3).
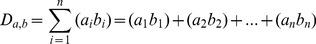
(3)Where *D_a,b_* is the dot product score for the pair of genes *a* and *b*, *n* is the total number of transcription factors interrogated in the study, and *a_i_* and *b_i_* are binding scores (often binary) for genes *a* and *b* and the *i^th^* transcription factor.

***ChIP followed by high-throughput sequencing (ChIP-seq) data.*** Transcription factor binding site and gene association scores [44], based on the genomic location of the binding site closest to the transcription start site of expressed genes, were used as raw data and processed in the same manner as ChIP-Chip data.

***Whole genome small interfering RNA (siRNA) screen data.*** Raw siRNA data from primary screens represented the percent of differentiating cells upon exposure to siRNA knockdown of mESC self-renewal genes. We organized these values into a matrix format analogous to that used to preprocess microarray and ChIP data. A Euclidean distance measure was used to distill this data into pairwise similarity scores (Equation 4).
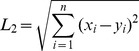
(4)Where *L_2_* is the Euclidean distance function calculated between mESC differentiation status levels when genes *x* and *y* are knocked down.

#### Preparation of curated, tissue-specific, training gold standard

We developed a comprehensive positive gold standard for mESC genes and gene products involved in self-renewal, curated from a literature library of 98 recent articles related to mESC self-renewal, pluripotency, and cell fate determination (Table S1). This library was supplemented with mouse developmental pathway information from KEGG [25]. A list of all publications referenced in the mESC gold standard is available in Table S2. From this mESC reference, we extracted a list of 2056 “positive pairs” of gene or gene products experimentally validated to be functionally related in the context of mESC fate. To generate a negative gold standard, we developed a list of 21,291 protein-coding genes (Table S12) derived from an MGI Sequence Coordinates report (MGI_Coordinate.rpt for Build 37, downloaded December 23, 2010) by selecting only MGI IDs that fit the following criteria: Marker Type – Gene, mapped to specific NCBI Gene Start and Stop coordinates, with an official symbol not prefaced by GM (for predicted Gene Marker), and for which at least one evidential dataset contained measurements. This gene list was used to randomly generate a list of “negative pairs” (excluding positive pairs) 10 times the size of the positive gene pair list. The resulting gold standard answer file (Table S13), consisting of a total of 22,616 gene pairs, was used as the prior knowledge to train the Bayes net.

### Construct Bayesian Network and Infer Posterior Functional Relationship Scores

To perform naïve Bayesian network machine learning techniques, we computed the posterior probability of a functional relationship between gold standard gene/protein pairs given all evidential data [17], [19], [66], [67]. We used the Sleipnir library of C++ tools for machine learning over genomic data [66] and the Structural Modeling, Inference, and Learning Engine (SMILE) C++ library, developed at the University of Pittsburgh [68]. Additional procedural details on using Sleipnir tools for data integration and network inference are provided in the Supplemental Notes (File S1).

#### Bayesian network training and inference

Conditional probability tables (CPTs) for each dataset were learned by counting the observed values in each dataset’s discretized bins for unrelated and related training gene pairs [10], [19]. Once learned, these CPTs were used to infer posterior functional relationship scores between pairs of genes or gene products. The posterior probability that two protein-coding genes participate in a self-renewal related biological process, given existing data, was calculated based on the prior probability of a functional relationship between genes and the conditional probability of observing evidential data given functional relationship status (Equation 5) [10], [67].
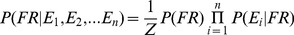
(5)Where *FR* is a hidden variable representing whether a gene pair is functionally related, *P(FR = 1)* is the predicted probability that a pair is functionally related, *E_i_* represents the evidence score of the gene pair for the *i^th^* dataset, and *Z* is a normalization factor.

#### Minimization of network overfitting

We performed four-fold cross-validation (on both edges and gene in the gold standard) and leave-one-gene out cross-validation (also called “jack knifing”) experiments to determine classifier performance and generality. To divide the training set into folds, we used two schemes: the first, randomly separating edges into sets, regardless of which genes were involved in those edges; the second, randomly eliminating a quarter of genes in the genome (more specifically the protein coding gene list) by removing all edges in a training set fold that contained those genes. For four-fold edge cross validation, we partitioned the gold standard edges into four randomly generated test sets, while preserving the 1∶10 class distribution (positive:negative ratio). We trained classifiers on three folds of the gold standard, using the withheld fold as a validation set, repeating this process four times so that all gold standard training edges were used for both training and validation, and each test fold was used for validation once. We conducted four-fold gene cross validation in a similar manner by partitioning the list of 21,291 protein-coding mouse genes into four gene folds, then creating four test sets, each including only edges involving genes within one fold, while corresponding training sets included edges only between genes in the remaining three folds. For leave-one-gene-out cross validation, we removed one well-known self-renewal gene (Lif, Nanog, Pouf51, or Sox2) and all edges involving that gene to create a test set, while all remaining genes were used for training.

To minimize overfitting, we performed Bootstrap aggregation (*i.e.* bagging) by 1) creating a series of 20 training and test gold standard files, each consisting of a random 70–30% split of the gold standard file, 2) performing bootstrap runs using these gold standard files (the number of bootstrap runs was determined by the point at which the network performance leveled off), and 3) averaging the inference scores for each gene pair across all bootstrapped networks for which the pair was not used as a training example (*i.e.* “out of bag” averaging) [37].

#### Feature set selection

To assess the importance of input data feature set selection, we compiled three additional libraries of integrated high-throughput mouse data: 1) a minimal feature set consisting of 16 datasets composed of non-tissue specific expression data and information downloaded from online genomic data resources similar to feature sets used for prior efforts (Table S5); 2) a negative control set of 646 datasets excluding mESCS and not specific to any tissue type (Table S6); and 3) a superset of features (labeled Mmu – an organism code for Mus musculus) consisting of all the data used to train our mESC-only network plus all data from the minimalist and negative control compendiums (Table S7) [17], [18], [20]. We performed a full set of performance evaluations, cross-validation, bootstrapping, and out of bag averaging on networks produced using each of these feature sets. We used these alternative networks to compare and contrast network topology and underlying biological meaning of inferred functional relationship scores.

#### Regularization

Bayes nets impose a strict assumption of independence between input data that is likely violated by many of our input datasets. This limitation can be largely mitigated through regularization of parameters to down weight the contribution of datasets with redundant information (Figure S2). Parameter regularization was performed using mutual information between datasets to weight the strength of prior belief for each dataset [19], [36]. Because the same subset of information could be shared many times among tissue- and context-specific datasets, this regularization provided a quantitative estimate of the amount of redundant information contained in each dataset as compared to all other datasets in the compendium. We calculated a heuristic sum of mutual information relative to the Shannon entropy of each dataset [69](Equation 6), exponentially decreasing the weight of a dataset as the amount of shared information increased and incorporated these values into the formula for calculating posterior probability (Equation 7) as previously described [10], [67].
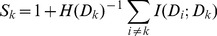
(6)

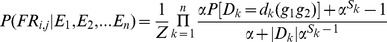
(7)Where *S_k_* is a heuristic sum of shared information relative to the dataset’s entropy used to weight the strength of prior belief in a uniform distribution for the dataset, *H* refers to Shannon entropy, and *I(D_i_;D_k_)* refers to mutual information. Equation 7 is an variation on Equation 5, such that *P(FR_ij_|E_1_, E_2_,…E_n_)* is the predicted probability that there is a functional relationship between genes *i* and *j* given evidence in datasets *1* through *n*, *Z* is a normalization factor, *α* is a pseudocount regularization parameter used to modulate the strength of regularization required as implied by the strength of the prior (higher pseudocount values weaken influence of redundant datasets), and *D_k_* is the number of bins used to discretize continuous data values in dataset K. A low S_k_ indicated the information contained in the dataset is highly unique, while a high score indicated the datasets contained shared (redundant) information. The redundancy score for each mESC dataset used to train the Bayesian classifier is listed in Table S1. We conducted a series of performance tests, evaluating effects of regularization on similarity score distributions in each evidential dataset and classifier performance, and selected an optimal pseudocount value of 70, which best fit our mESC training set. To produce a similar distribution of posterior edge values for the superset (Figure S2B) and negative control, we used a pseudocount value of 10.

### Computationally Test and Validate Results

We validated the accuracy of predicted functional relationships computationally using standard machine learning metrics and accepted protocols.

#### Evaluation metrics

To assess network predictive accuracy, we used standard statistical performance measures for binary (true/false) classification tests: Receiver Operating Characteristic (ROC) Curves, Area Under the ROC Curve (AUC), Precision-Recall Curves (PRC), and Area Under the PRC (AUPRC) [7], [10].

A ROC curve is a two-dimensional graph of true positive rate (TPR) versus false positive rate (FPR) (Equations 8, 9) that illustrates the relative tradeoff between benefits (true positives, TPs) and costs (false positives, FPs). Precision-recall (PR) curves depict the tradeoff between precision, which is a measure of exactness or quality (i.e. how many positive claims are correct), and Recall, which is a measure of completeness or quantity (i.e. how many positives were claimed of all possible positives) (Equations 10, 11) [54].
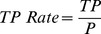
(8)

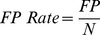
(9)

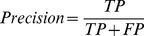
(10)

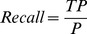
(11)


To ensure that network inferences were robust and to assess any evidence of overfitting, we performed four-fold gold standard and genome cross validation, leave-out-one cross validation, and bootstrapping (for details, see “Minimization of Network Overfitting”).

#### Gold standard evaluation

To assess the importance of training the Bayes net using a curated, cell-type-specific gold standard, we generated a series of test gold standards consisting of randomly generated negative pairs and positive genes pairs automatically generated from lists of genes associated with GO terms specific to self-renewal (stem cell maintenance - GO:0019827) and independent of stem cell self-renewal (cellular response to insulin stimulus - GO:0032869, regulation of cardiac contraction - GO:0008016). In addition, we created three gold standards of positive and negative gene pairs randomly generated from our list of protein coding genes. We trained Bayes nets using these alternative gold standards and the same feature set of mESC data, and used the performance metrics described earlier to evaluate results.

#### Network topology analysis

To analyze the network topology and evaluate biological information contained within graph files, we calculated degree (k), sum of degrees (ki), mean degree (kmean), and scaled degree (Ki), for each gene in the training set (Equations 12–15) [70].
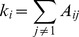
(12)

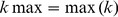
(13)

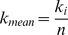
(14)

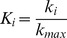
(15)Where the degree of the *i* ^th^ node of vector *k* (k_i_) equals the sum of edge weights between node *i* and all other nodes in the training set, and Adjacency matrix *A_ij_* quantifies the connection strength from node *i* to node *j*; the mean degree (*k_mean_*) is the degree *k_i_* divided by the total number of nodes *n* in the training set; and *k_max_* is the maximum degree across all *n* components of vector *k*.

#### Functional correlation scores

For functionally directed analyses, we calculated a functional correlation score Si, for a gene i as the average edge weight between gene i and all genes within a functional set of genes G within a network represented by adjacency matrix Aig (Equation 16).
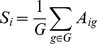
(16)


For our results, we created two sets of these scores. The first used the set of 354 genes in the positive gold standard to calculate functional correlation scores to the positive gold standard. Based on these scores, we observed that only a subset of our gold standard edges were strongly connected to the rest of the gold standard genes in our results. Therefore, we used an quasi-active-learning approach to refine our set to a highly correlated subset of self-renewal genes, those with an gold standard functional correlation score of 0.25 or higher (the top 52% of gold standard genes ranked by functional correlation score). Using this subset of 189 strongly correlated genes, we calculated and scaled an updated functional correlation score to this “golden” gold standard set of known self-renewal genes. We refer to this measure as the self-renewal correlation (SRC) score.

We used these values to identify major gene hubs within networks, and segregate clusters of genes that shared similar network properties.

## Additional Resources

Database for Annotation, Visualization, and Integrated Discovery (DAVID), http://david.abcc.ncifcrf.gov/.

Graph Algorithms Pipeline for Pathway Analysis (GrAPPA), http://grappa.eecs.utk.edu/.

Mouse Genome Informatics, http://www.informatics.jax.org.

Sleipnir Library for Computational Functional Genomics, http://huttenhower.sph.harvard.edu/sleipnir/index.html.

Structural Modeling, Inference, and Learning Engine (SMILE), http://genie.sis.pitt.edu/.

Thinkmap Visualization Technology, http://thinkmap.com.

Weighted Correlation Network Analysis (WGCNA), http://www.genetics.ucla.edu/labs/horvath/CoexpressionNetwork/Rpackages/WGCNA/.

The following data files are available online at StemSight.org/stemdata.html: mESC Network Graph File (∼18.1 million edges, edge weight ≥0.2), Datasets Supporting Top 0.01% of mESC Network Edges (∼226 thousand edges), Mmu Superset Network Graph File (∼7.8 million edges, edge weight ≥0.2), Negative Control Network Graph File (∼13.6 million edges, edge weight ≥0.2), Minimalist Set Network Graph File (∼4.3 million edges, edge weight ≥0.2).

## Supporting Information

Figure S1 **mESC Network Performance Evaluation through Cross Validation.**
(DOCX)

Figure S2 **Effect of Regularization on mESC and Test Networks.**
(DOCX)

Figure S3 **Dataset Classes Supporting Top mESC Network Edges.**
(DOCX)

Figure S4 **Data Compendium Tests.**
(DOCX)

Figure S5 **Datasets Supporting** ***Tdh*** **Connectivity to Gold Standard Genes.**
(DOCX)

Table S1 **mESC Self-Renewal Gold Standard Positive Edges and Supporting Literature References.**
(XLSX)

Table S2 **Journal Articles Referenced to Curate mESC Gold Standard.**
(XLSX)

Table S3 **mESC Data Compendium.**
(XLSX)

Table S4 **mESC Network Gene Ontology Annotation Enrichment.**
(XLSX)

Table S5 **Minimalist Data Compendium.**
(XLSX)

Table S6 **Negative Control Data Compendium.**
(XLSX)

Table S7 **Superset Data Compendium.**
(XLSX)

Table S8 **Data Compendium Tests.**
(XLSX)

Table S9 **Network Topology Statistics for mESC and Mmu Networks.**
(XLSX)

Table S10 **Gold Standard Posterior Edge Weight and Rank Order.**
(XLSX)

Table S11 **Datasets Supporting Top Ranked mESC Network Edges.**
(XLSX)

Table S12 **Protein Coding Gene List.**
(XLSX)

Table S13 **mESC Gold Standard Answer File.**
(XLSX)

Appendix S1 **Gene and Signaling Pathway Reference.**
(XLSX)

File S1 Notes. Protocol for Preprocessing Microarray Datasets and Using Sleipnir Tools.
(DOCX)
