# Supplementary material for: Cell-Type-Specific Predictive Network Yields Novel Insights into Mouse Embryonic Stem Cell Self-Renewal and Cell Fate
Source: PLoS One. 2013 Feb 28;8(2):e56810. doi: 10.1371/journal.pone.0056810 (PMC3585227; doi:10.1371/journal.pone.0056810)
Supplement: Figure S1 — mESC Network Performance Evaluation through Cross Validation. (DOCX) [file pone.0056810.s001.docx]

**
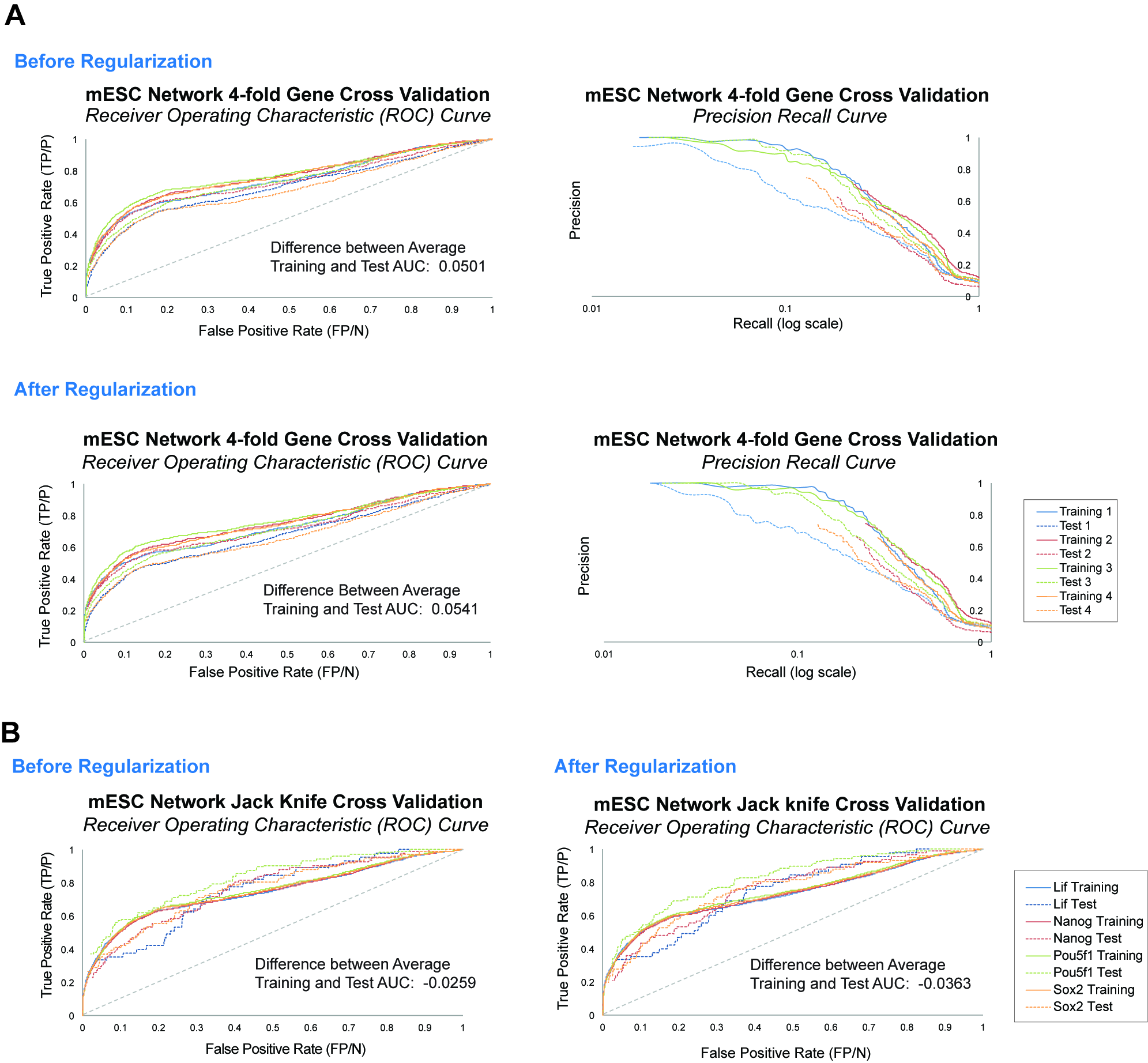
**

**Supplemental Figure S1.** **mESC Network Performance Evaluation through Cross Validation. A.** 4-Fold Gene Cross Validation. In addition to performance evaluations presented in Figure 3, we conducted 4-fold gene cross validation by removing 25 percent of genes in the gold standard training set. We first divided our list of 21,291 protein coding genes into four sub-lists, then used these lists to create four set of test (25 percent) and training (75 percent) files. Regularization reduced some overfitting; the remainder was largely corrected for through out-of-bag averaging.
**B.** Leave-One-Gene-Out Cross Validation. We performed a variation on Jack Knife cross validation by removing edges containing a gene known to be essential for mESC self-renewal and pluripotency: *Lif, Nanog, Pou5f1, and Sox2.* Comparison of performance results before and after regularization illustrate the ability of the Bayes net to learn even when examples that included these key self-renewal genes were removed from the training set.
