# Supplementary material for: Cell-Type-Specific Predictive Network Yields Novel Insights into Mouse Embryonic Stem Cell Self-Renewal and Cell Fate
Source: PLoS One. 2013 Feb 28;8(2):e56810. doi: 10.1371/journal.pone.0056810 (PMC3585227; doi:10.1371/journal.pone.0056810)
Supplement: Figure S2 — Effect of Regularization on mESC and Test Networks. (DOCX) [file pone.0056810.s002.docx]

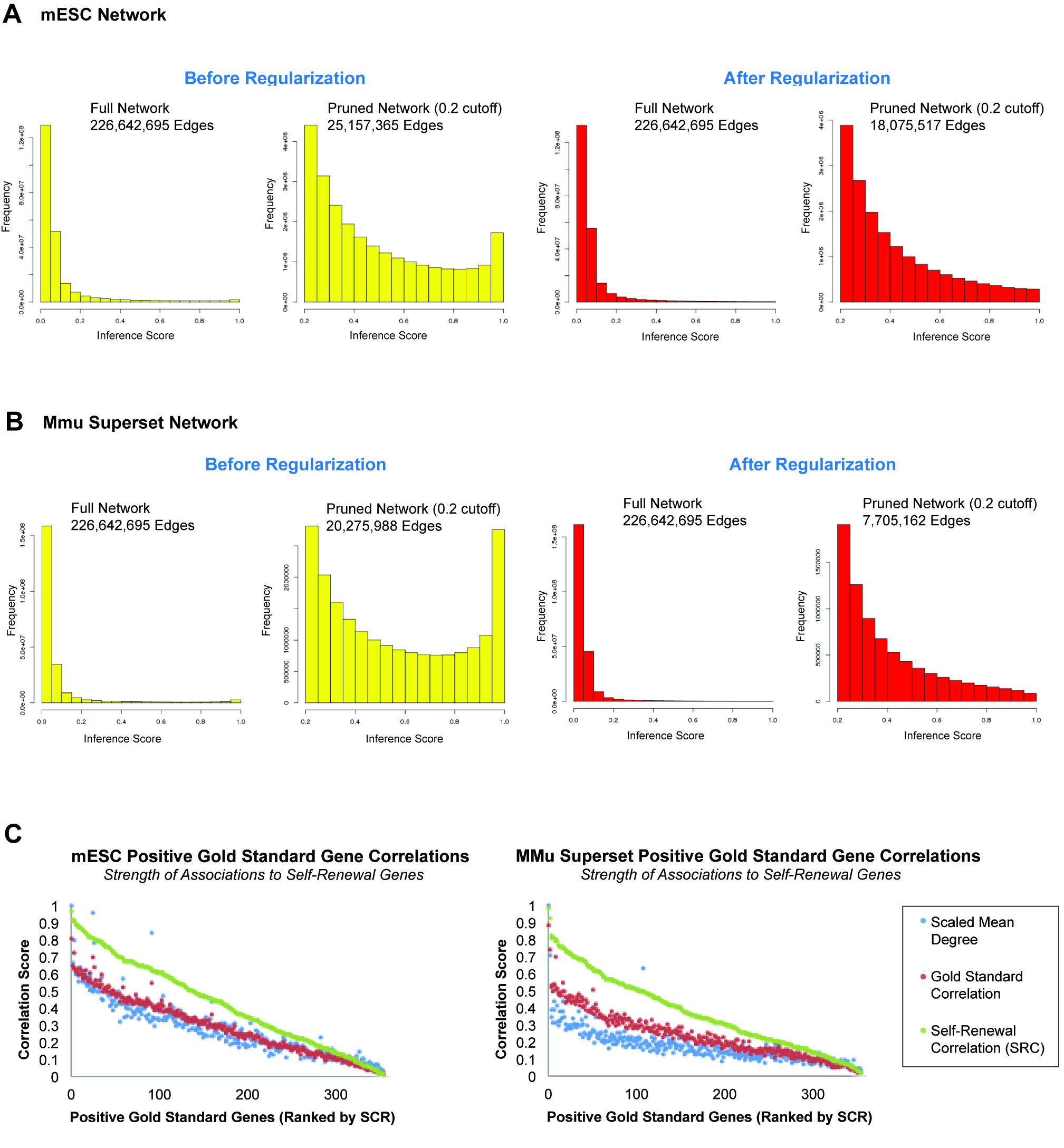


**Supplemental Figure S2. A, B. Effect of Regularization on mESC and Test Networks. C. Self-Renewal Connectivity Strength of Gold Standard Genes.** Regularization was performed to account for redundant information in large data compendiums. We adjusted regularization parameters to achieve a similar edge weight distribution pattern in both mESC-only and Mmu superset networks (see Material and Methods for details). **A.** Prior to regularization (yellow distributions), the mESC network had ~25 million edges with an inferred edge weight greater than 0.2, and a notable increase in edges near 1.0. After regularization (red distributions) to reduce the impact of redundant information, the number of edges with an inference score greater than 0.2 decreased to ~18 million. This process served to reduce the pile-up among edges with posterior edge weights at or near 1.0 caused by redundant evidence. **B.** The superset network before regularization had ~20 million edges with an inference score greater than 0.2 and a very sharp increase in edges near 1.0. After regularization, the superset network had only ~7.7 million edges with an inference score greater than 0.2. **C.** Comparisons of mESC and superset network positive gold standard gene correlations. The self-renewal correlation (SRC) score is a “guilt by association” measure of the strength of functional linkage between a given gene and most highly connected genes in the mESC positive gold standard, those with a gold standard functional correlation greater than 0.25. This SRC value (shown in green) is closely related to scaled degree (blue) and gold standard correlation (red), but gives a purer measure of correlation without noise introduced by incomplete biological knowledge. This distribution of the gold standard edges shows that some edges are more supported by the evidential data than others.
