# Supplementary material for: Cell-Type-Specific Predictive Network Yields Novel Insights into Mouse Embryonic Stem Cell Self-Renewal and Cell Fate
Source: PLoS One. 2013 Feb 28;8(2):e56810. doi: 10.1371/journal.pone.0056810 (PMC3585227; doi:10.1371/journal.pone.0056810)
Supplement: Figure S3 — Dataset Classes Supporting Top mESC Network Edges. (DOCX) [file pone.0056810.s003.docx]

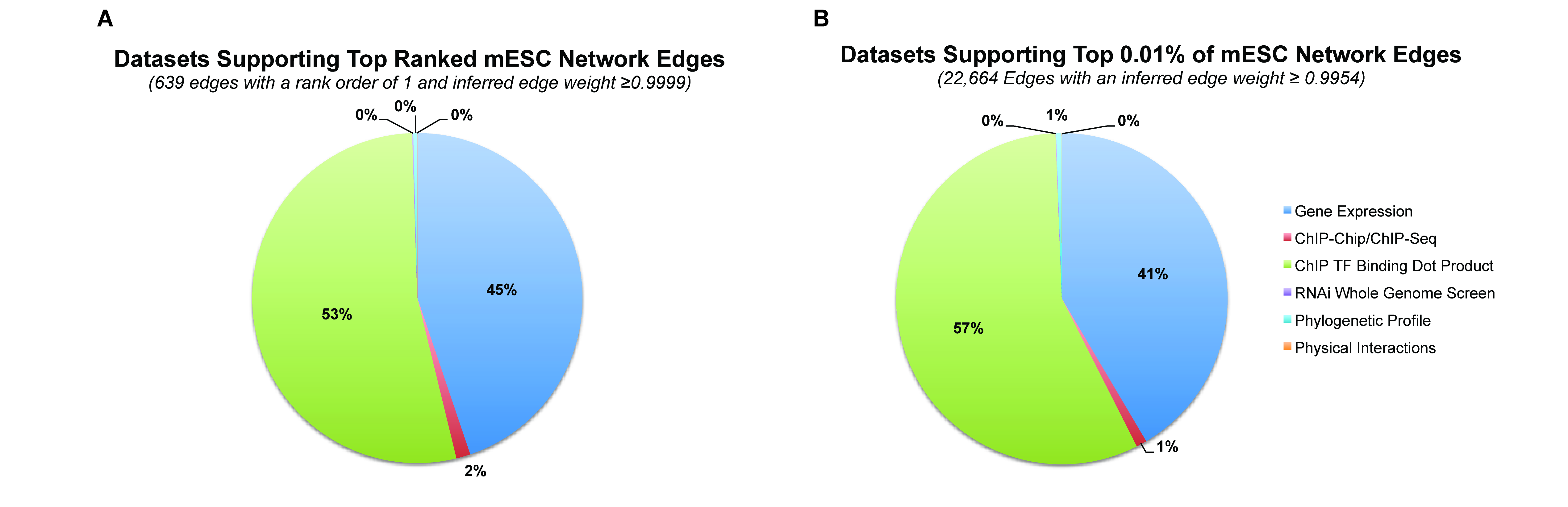


**Supplemental Figure S3. Dataset Classes Supporting Top mESC Network Edges.** High-confidence edges in the mESC network are supported by a diverse set of high-throughput data, illustrating that to achieve the best network performance in terms of biological relevance, data from many different types of experiments should be included in the data compendium. The Bayes net found Protein-DNA binding similarity profiles, followed by gene expression data, the most informative. Molecular interaction data downloaded from online resources and raw RNAi data were the least informative, largely due to the limited scope of these data. Supplemental Table S12 provides more details on the individual datasets that support the top 0.01% of network edges. **A.** The 639 top ranked mESC edges, those with a rank order of 1 and inferred edge weight ≥ 0.9999, were largely supported by transcription factor binding similarity profiles (dot products) calculated using protein-DNA binding data from ChIP-Seq and ChIP-Chip studies. Our Bayes net finds protein-DNA datasets the most reliable and dot products between vectors of individual transcription factor binding scores provide much more information than individual ChIP-based arrays. **B.** The top 0.01% of edges, 22,664 edges with an inferred weight ≥ 0.9954 were supported by a slightly different distribution of high-throughput data, with a smaller percentage of gene expression datasets contributing to edge weight. These top ranked edges are predominantly supported by dot products from evidential data from 12 ChIP-based studies with arrays for well-characterized genes known to play a key regulatory role in mESCs.
