# Supplementary material for: Cell-Type-Specific Predictive Network Yields Novel Insights into Mouse Embryonic Stem Cell Self-Renewal and Cell Fate
Source: PLoS One. 2013 Feb 28;8(2):e56810. doi: 10.1371/journal.pone.0056810 (PMC3585227; doi:10.1371/journal.pone.0056810)
Supplement: Figure S4 — Data Compendium Tests. (DOCX) [file pone.0056810.s004.docx]

_
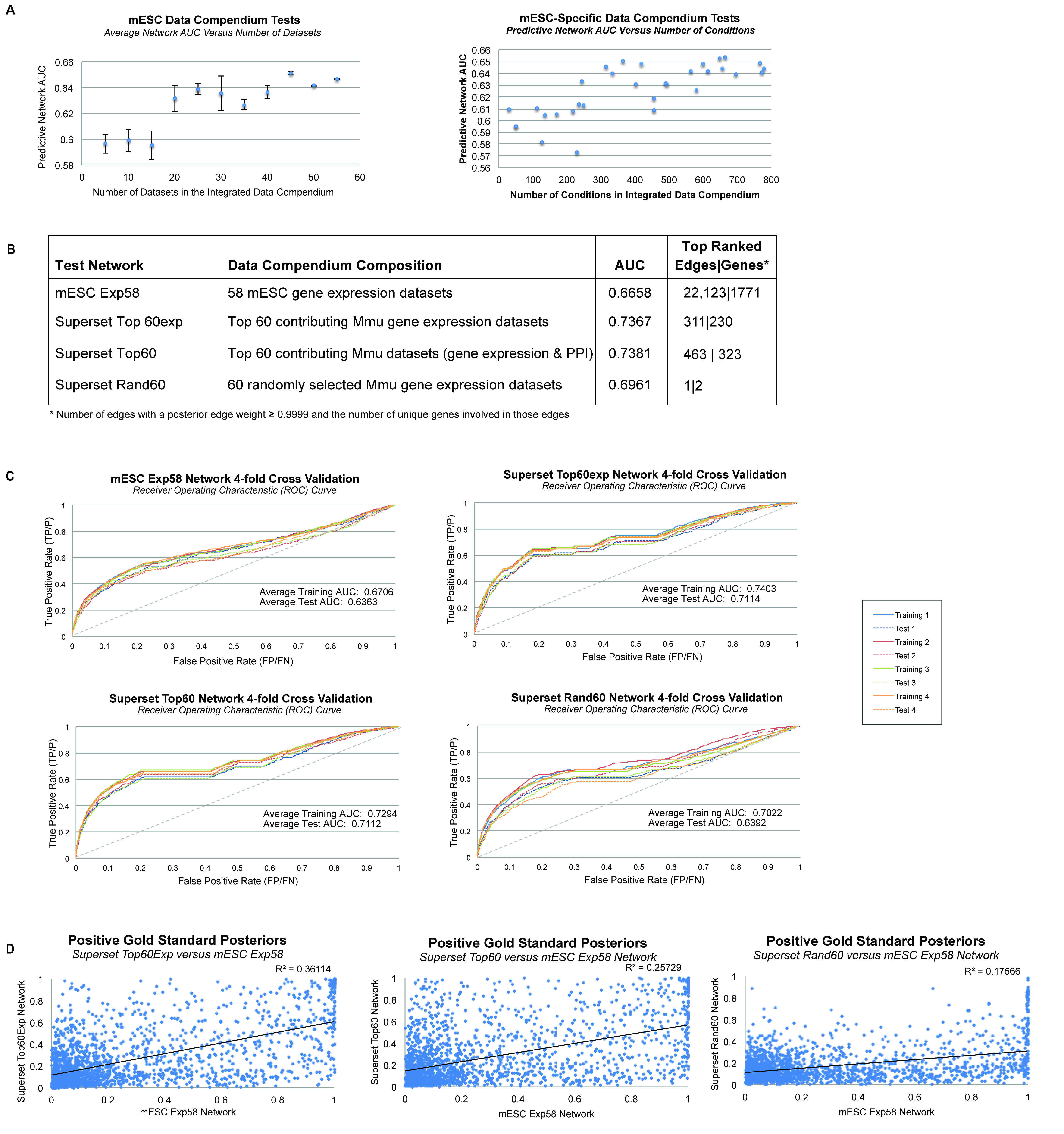
_

**Supplemental Figure S4. Data Compendium Tests. A.** To evaluate how compendium size contributed to the Bayes net’s ability to learn, we generated sets of test compendiums of different sizes (from 5 to 55 randomly selected datasets) using the 58 mESC gene expression datasets included in this study. AUCs plateaued at approximately 0.65 at ~45 datasets or ~600-700 conditions. **B.** To compare the effect of using “inappropriate” non-cell-type-specific data compendiums of roughly the same size as the mESC network, we created three additional test compendiums using Mmu superset data: the top 60 gene expression datasets (as ranked by the Bayes net), the top 60 gene expression and PPI datasets, and 60 randomly selected datasets. We generated networks using these compendiums and the same mESC self-renewal gold standard and master gene list. All three Mmu test networks achieved higher AUCs than the mESC expression only network, but contained fewer high-confidence top ranked edges with a posterior edge weight ≥ 0.9999. Only the mESC network had edges with a posterior edge weight of 1 that involved genes that were highly enriched for biological processes associated with stem cell maintenance, embryonic development, and cell cycle controls (Supplemental Table S13). These results underscored that computation performance metrics alone are insufficient for evaluating cell-type-specific networks. **C.** Four-fold gold standard edge cross validation of these networks showed evidence of mild overfitting in all test networks. The greatest overfitting was observed in the Mmu Random 60 network, the least in the Mmu Top 60 gene expression network. **D.** Correlation of gold standard edge weights showed that each of these networks had very different posterior edge weights, meaning the biological content of networks varied greatly. The mESC gene expression network was most similar to the Mmu Top 60 gene expression network (R^2^= 0.36), but there were stark differences. Many edges between developmental signaling pathway pairs, such as Fgfr3 – Araf and Bmpr1 – Mapk1, that were strongly supported in the Mmu Top 60 compendium, yet weakly supported in the mESC only network. Similarly edges involving canonical pluripotency factors, such as Pou5f1 – Cdkn1a and Nanog – Phc2, were strongly supported in the mESC network but not in the Mmu Top 60.
