# Supplementary material for: Cell-Type-Specific Predictive Network Yields Novel Insights into Mouse Embryonic Stem Cell Self-Renewal and Cell Fate
Source: PLoS One. 2013 Feb 28;8(2):e56810. doi: 10.1371/journal.pone.0056810 (PMC3585227; doi:10.1371/journal.pone.0056810)
Supplement: Figure S5 — Datasets Supporting Tdh Connectivity to Gold Standard Genes. (DOCX) [file pone.0056810.s005.docx]

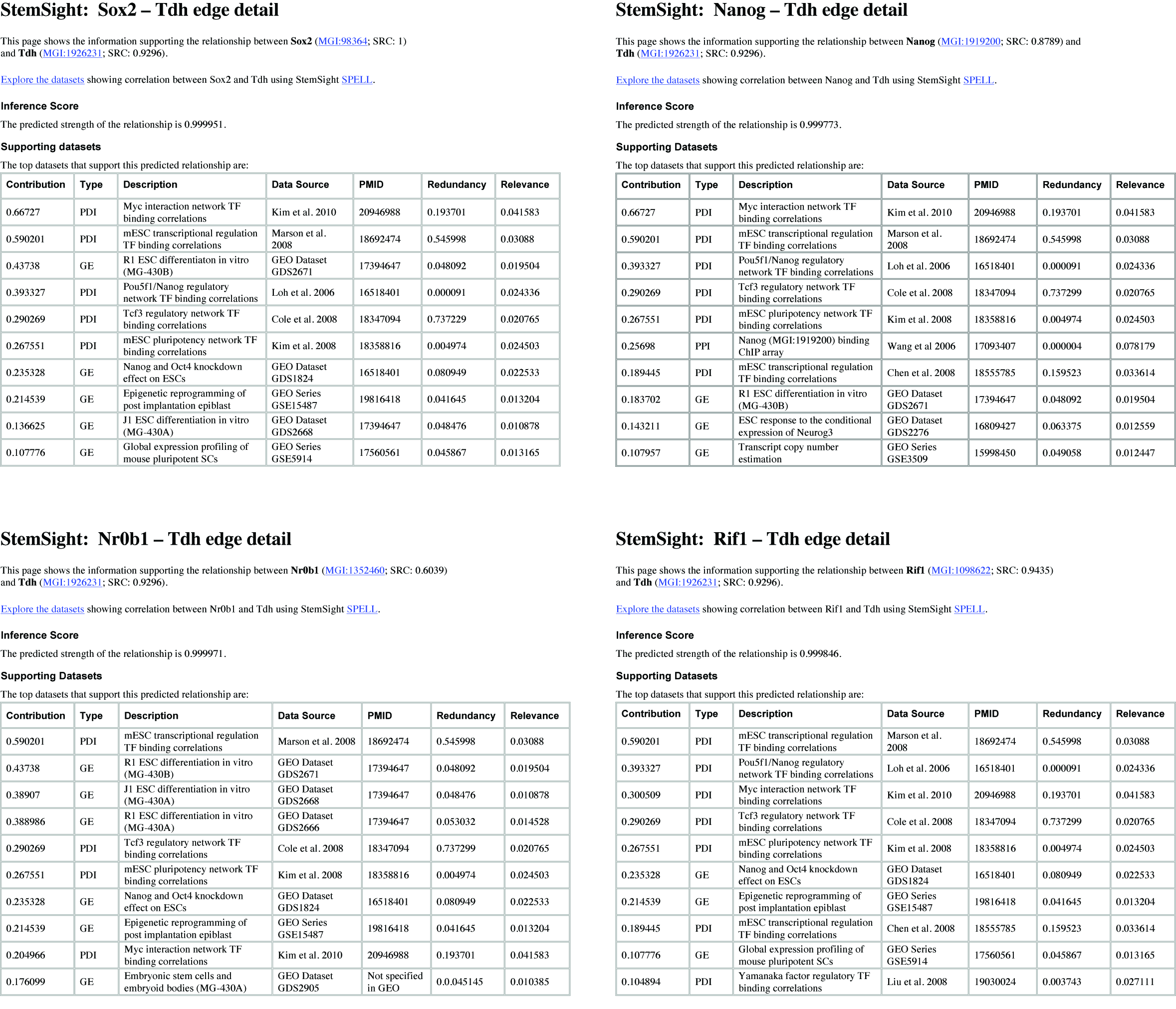


**Supplemental Figure S5. Datasets Supporting *Tdh* Connectivity to Gold Standard Genes.** The top 10 datasets supporting high-confidence edges between *Tdh* and gold standard genes *Sox2, Nanog, Nr0b1,* and *Rif1* are predominantly composed of transcription factor binding similarity profiles, suggesting that *Tdh* is likely to be a target of genes involved in the regulatory circuitry of pluripotency and self-renewal. This is consistent with recent evidence that mESCs have unusual, accelerated cell-cycle kinetics to support rapid growth phase of early embryonic development that require high amounts of ATP as fuel.
