# Supplementary material for: Cell-Type-Specific Predictive Network Yields Novel Insights into Mouse Embryonic Stem Cell Self-Renewal and Cell Fate
Source: PLoS One. 2013 Feb 28;8(2):e56810. doi: 10.1371/journal.pone.0056810 (PMC3585227; doi:10.1371/journal.pone.0056810)
Supplement: File S1 — Notes. Protocol for Preprocessing Microarray Datasets and Using Sleipnir Tools. (DOCX) [file pone.0056810.s020.docx]

# Supplemental Notes

These supplemental notes contain step-by-step instructions for preprocessing microarray gene expression data to use as evidential data and for generating cell-type-specific naïve Bayesian networks using C++ tools from the Sleipnir Library of Computational Functional Genomics (http://www.huttenhower.org/sleipnir/index.html). The ruby scripts mentioned in this documentation are available on request from the Hibbs Laboratory StemSight team (email stemsight@jax.org.) Specifically, this document covers:

Gene Expression Data Pipeline 2

Initial data collection 2

For Gene Expression Omnibus (GEO) dataset files 2

Download all GDS datasets from GEO 2

Collect information from all datasets and convert to pcl format 2

For GEO Series (GSE) Files 3

Perform custom download of specific GSE files of interest 3

Manually curate GSE files to prepare for pipeline processing 3

Convert GSE files into PCL format 3

Generate Info file for all GSE PCL files to be processed in pipeline 3

Data Normalization Pipeline 3

Insert missing values 3

Impute missing values and remove probes with few values present 4

Map probes to genes 4

Average together duplicate genes 4

Final numeric clean up and consolidation 4

Using Sleipnir Tools to Generate Cell-Type-Specific Predictive Networks 5

What you need 5

Prepare Network Directories and Input Files 5

Create Gold Standard Answer File 6

Convert dataset pcl files to .dab format 6

Verify .dab files were converted and transformed properly 7

Create corresponding quant files for each dataset .dab file 7

Double-check working directories and files 7

Create Networks 8

Generate conditional probability table (CPT) counts (Counter –w Step) 8

Produce a dataset list 9

Generate CPTs (Counter –k Step) 9

Make predictions (Counter –n Step) 10

Analyze Bayes Net Performance 11

## Gene Expression Data Pipeline

### Initial data collection

This will vary from source to source, but the output of the first phase is always a preclustered (PCL) formatted file and meta information about the data.

#### For Gene Expression Omnibus (GEO) dataset files

To collect curated GEO dataset (GDS) files:

Download all GDS datasets from GEO

By anonymous logon to the NCBI ftp site, all available microarray data can be downloaded from <ftp://ftp.ncbi.nih.gov/pub/geo/DATA/SOFT/GDS/> as gzipped soft formatted files. Currently the easiest way to get all data for a single organism is to download all of the available data and verify organism after reading out header information.

Collect information from all datasets and convert to pcl format

We can identify information about each dataset, such as organism, platform used, sample descriptions, etc. from the header information in the soft files. We’ll do this at the same time as we read the files to convert them to pcl format. To do this, run the ruby script **allSoft2Pcl.rb** and store the output:

ruby allSoft2Pcl.rb <path_to_soft_files> > <info_file>

This will create an info file that contains basic information about each file, as well as creating a pcl formatted file for every *.soft file in the specified directory. Alternately, files can be converted one at a time using the script **convertSoft2Pcl.rb.**

In a properly formatted pcl file, there are two header lines, the first two columns are probe IDs or identifiers, followed by a weight column, then values for specific arrays. It should be a tab-delimited files that looks like this:

YORF NAME GWEIGHT <Array1> <Array2> <Array3>…

EWEIGHT 1 1 1 1 1

<Gene1> <Gene1> 1 <value1.1> <value2.1> <value3.1>

<Gene2> <Gene2> 1 <value1.2> <value2.2> <value3.2>

<Gene3> <Gene3> 1 <value1.3> <value2.3> <value3.3>

.
.
.

#### For GEO Series Files

GEO Series (GSE) Files have been uploaded to the GEO repository by researchers, but have not by curated by through GEO. These are the source files for curated GDS datasets, so be careful you don’t use both the GDS and GSE for the same study. In some cases, there is a problem with the uploaded GSE files that prevent it from passing GEO curation and there is no corresponding GDS file.

Perform custom download of specific GSE files of interest

This requires more customized searches on the NCBI GEO site (<http://www.ncbi.nlm.nih.gov/geo/>) to identify datasets that contain microarray data of interest. Identify key words that will retrieve all potential GSE files of interest.

Manually curate GSE files to prepare for pipeline processing

Using a text editor, such as Gnu EMACS or VI, remove any arrays that are not specific to your study. If more than one platform is used, split the series file into two or more files, one for each platform

Convert GSE files into PCL format

GSE files are all “special” in that they all have (usually minor) problems with the file format or content that causes them not to be automatically curated into GDS files. For that reason, you’ll need to do some extra work to prepare the GSE family.soft files for processing in our pipeline. Use the seriesFamilyParser.rb script:

ruby seriesFamilyParser.rb <filename> > <filename.pcl>

This script will prompt you to answer a series of questions about the microarray data to identify which column contains the probeID, gene name (symbol), values, whether the data has one or two channels, if it’s been log transformed, etc. It will then generate an output file in pcl format

Generate Info file for all GSE PCL files to be processed in pipeline

Ensure all pcl files generated by the seriesFamilyParser script are in the same directory. Create an InfoFile that contains general reference information about all pcl files that will be used in subsequent pipeline processing steps. To do this, run the **AllPcl2Info.rb** script:

ruby AllPcl2Info.rb <path to pcl files > > <info_file>

Other data repositories and sources used by the preprocessing steps vary. Any files to be used as evidential data must be in a PCL matrix format.

### Data Normalization Pipeline

Insert missing values

While non-numeric entries will have already been turned into missing values, some laboratories use values that are better characterized as missing. Run the script **insertMissingValues.rb** to address these:

ruby insertMissingValues.rb <pcl_file> <info_file>

This script will append .mv to the pcl file as the newly created output.

Impute missing values and remove probes with few values present

Since later methods require a full data matrix, we use KNN-Impute to fill in missing values with their most likely values. However, this process is unreliable when few actual values are present, so probes are required to be present in at least 70% of the conditions to be kept. Run the script **runKnnImpute.rb** to do this:

ruby runKnnImpute.rb <pcl_file> <info_file> <path_to_KNNImputer>

This script will run **KNNImputer** with the proper parameters and pre/post-processing, and append .knn to the pcl file as the newly created output. KNNImputer is part of the Sleipnir library (Huttenhower et al., 2008).

Map probes to genes

Using the script **mapGeneNames.rb**, potential aliases and conflicts are resolved by translating all gene names to a common standard. This script requires a file that includes the organisms of interest and points to alias mapping files. These files should contain 2 tab-delimited columns, the 1^st^ of which contains single aliases, and the 2^nd^ of which contains standard names that are | (bar)-delimited. This script can be run with:

ruby mapGeneNames.rb <pcl_file> <info_file> <org_file>

This will append .map to the newly created output, if the organism of the file is included in the script hard-coded list. Otherwise, no new output file is made.

Average together duplicate genes

The Java program **MeanGenesThatAgree.jar** will calculate the mean of all probes that meet a maximum likelihood test of measuring the same gene. Specifically, this method compares the distribution of Euclidean distances between pairs of probes annotated to the same gene to the distance distribution between randomly selected probe pairs. Probes are averaged together and annotated to a single gene if their distance is more likely to be drawn from the annotated probe distribution than from the random distribution. This script can be run with:

java –Xmx2g –jar MeanGenesThatAgree.jar <pcl_file> 1 > <out_file>

Name the outfile to append .avg to the newly created output file.

Final numeric clean up and consolidation

Some data files require a final numerical transformation (logarithm of all measurements, performed with a call to **DivLogNorm.jar).** This script performs this if necessary, and always moves the final file to an organism specific directory:

ruby collectFinalData.rb <pcl_file> <info_file> <script_path> <output_path>

*Note:* There is also a script that runs the entire pipeline for a single input pcl. It can be run with:

ruby fullyNormalizeDataset.rb <pcl_file> <info_file> <org_file> <path_to_pipeline_scripts> <path_to_KNNImputer> <output_path> [REMAKE]

## Using Sleipnir Tools to Generate Cell-Type-Specific Predictive Networks

These are the basic steps for using Sleipnir tools to generate cell-type-specific predictive networks. You will need to install the Sleipnir library of tools on your server before you can begin. The computational resources and time required to use these tools varies depending on the size of your data compendium, gold standard, and master gene list. On the Hibbs Lab cluster (~250 compute cores), the mESC network, with 164 evidential datasets, takes ~4 hours to compute. In contrast, the superset network, with 810 datasets, takes ~16 hours. Cross validation and bagging is more time-consuming and the total time required depends on the number of bootstrap runs required. In general, it takes about 2 weeks to perform regularization and bootstrap aggregation on a final network and to prepare the supporting files required to publish the network online in our visualization tool.

### What you need

- Gold Standard positive and negative lists of gene pairs (all genes should be referenced using consistent IDs, *e.g.* MGI:ID, Entrezgene ID, official gene symbol)
- Two gene lists of all protein-coding genes in your genome of interest, one numbered.
- Optional: Context gene list(s) of all genes within a biological context of interest
- Optional: Context file that lists all context gene lists to be used
- Datasets in .pcl format
- Sleipnir tools: Distancer, Dat2Dab, Counter, DChecker. For installation instructions, go to <http://libsleipnir.bitbucket.org/>
- R
- Microsoft Excel

### Prepare Network Directories and Input Files

To generate naïve Bayesian Networks using integrated genomic data, you will need a gold standard training set and a list of protein coding genes and directory containing evidential data files. Because there are several steps that generate intermediate files, create a directory structure to organize these files for each network, such as:

**Directory Name Description**

Network Network folder

dabs Integrated cell-type-specific data .dabs and .quants file folder

gl Gene list folder

gs Gold standard folder

counts Conditional probability table counts folder

results Prediction results folder

xval Cross validation folder

analysis Network analysis folder

Create Gold Standard Answer File

Screen your positive and negative gene pair lists to ensure there are no duplicate gene pairs in these lists (*i.e.* no positive pairs should appear in the negative list). Combine your positive and negative gene lists to create one tab-delimited text file, with three columns. The format should be:

**Relationship Type Answer File Format**

positive gene pair MGI ID <tab> MGI ID 1

negative gene pair MGI ID <tab> MGI ID 0

Once you have an answer file that contains positive and negative gene pairs and their respective relationship values, convert the file to .dab format, which is a compressed file format that can be read and processed by Sleipnir tools. Use **Dat2Dab** to convert a text file with Unix (lf) line breaks to .dab format. Depending on the directory path to your installed Sleipnir Tools, the basic command is:

Dat2Dab –i <answers.dat> –o <answers.dab>

Where <answers.dat> is the name of your *input* file and <answers.dab> is your *output* file. Use this same tool to convert .dab files to readable text format.

Even though your answer file should have only two classes (0,1), you need to create a file that defines answer file bins for the Bayes net. To do this, create a corresponding quantification (.quant) file for every .dab file you generate. This is simply a tab-delimited text file that contains one line that specifies the bins that will be used to discretize continuous data. For the answer file, which has only two values (0 and 1), there are only two bins:

0 <tab> 1

You can create this file in any text editor. The file should have the same name as the .dab file, with a .quant extension. For example:

Answer File: gs_answers.dab
Answer Quant File: gs_answers.quant

Save these two files (.dab and .quant) to your working Bayesnet directory. For more information on Dat2Dab options, see <http://huttenhower.sph.harvard.edu/sleipnir/Dat2Dab.html>.

Convert dataset pcl files to .dab format

Run **Distancer** to convert pcl files to .dab format that can be processed by Sleipnir. These .dab files contain pairwise similarity score calculated from the microarray data in the .pcl files. The basic command is:

Distancer –i <dataset1.pcl> -o <dataset1.dab>

Where *dataset1.pcl* is the name of the *input* file you want to convert and *data.dab* is the name of the *output* file you’ll use when generating Bayes nets with Sleipnir tools. Save these evidential data .dab files to a dabs/ subdirectory within your working network directory. If you have multiple data compendiums, create a separate subdirectory for each (for example: dabs_mESC/, dabs_superset/, dabs_minset/).

*Note:* The default **Distancer** command produces a z-score (z-transformed Pearson correlations) for each gene pair in the .pcl file. If you want to test other similarity measures, add a -d switch to the command line. Valid Distancer -d values are: "pearson", "euclidean", "kendalls", "kolm-smir", "spearman", “pearnorm", "hypergeom", "innerprod", "bininnerprod", "quickpear", "mutinfo", "relauc", "pearsig". The default is "pearnorm".

For more information on Distancer options, see <http://huttenhower.sph.harvard.edu/sleipnir/Distancer.html>.

Verify .dab files were converted and transformed properly

Use R to check the normalization curves of the data in the .dab files created by **Distancer**. The .dab files are not in a format R can read, so you must first convert them back to tab-delimited data files (.dat files).

Run **Dat2Dab** to create a set of .dat files from your newly created .dab files. The basic Dat2Dab command is:

Dat2Dab –i <dataset1.dab> - o <dataset1.dat>

Where dataset1.dab is the name of your machine-readable input file and dataset1.dat is the text outfile. If you used the default Pearson Normal Distancer option to produce evidential data .dab files, use R or your statistics program of choice to produce a histogram from the .dat file and check the distribution.

Verify the normalization curve looks good. If so, move the .dab file to a dabs/ subdirectory in your working directory. If the normalization curve don’t look “normal,” refer to the source dataset in your master file directory or repository and double-check the number of samples in the source datasets. Microarray datasets must have at least 4 samples to be processed correctly.

Note that .dat files generated from .dab files may be quite large. You may want to create a script that converts .dabs to dats, produces the R histogram, then deletes the intermediate .dats if disk space is limited.

Create corresponding quant files for each dataset .dab file

Expression values in high-throughput microarray data files are continuous. To discretize this data for the Bayesian classifier, you need to create quant files for each dataset in your <input_file> subdirectory. In our case, the quant file for all microarray datasets was a one-line, tab-delimited text file containing these seven bins:

-1.5 -0.5 0.5 1.5 2.5 3.5 4.5

Verify that you have one .quant file for every .dab file in your subdirectory and that all files use a consistent naming format. For example:

transfac.dab

transfac.quant

GSE9978.dab

GSE9978.quant

Double-check working directories and files

Verify that you have created dabs, counts, and results subdirectories for your network files, and that you have all the files you need to generate a Bayes net: your answer files (.dab and quant), two gene lists (one numbered), a optional context file (a subset of protein-coding genes, such as for a biological process or developmental stage), evidential dataset files in .dab format (in a separate dabs/ subdirectory with corresponding quant files).

*Note:* Most issues with generating Bayes nets using the Sleipnir Counter tool are due to missing files, misnamed files, errors in paths, *etc.* This checkpoint will help you avoid potential problems and unnecessary angst.

### Create Networks

Generate conditional probability table (CPT) counts (Counter –w Step)

Use Sleipnir **Counter** to generate a counts file in your output directory. This counts file summarizes the number of data values in the each discretized dataset.dab file relative to the functional gold standard in the answer file. To generate global counts, use the following Counter command:

Counter –w <answers.dab> -d <dabs/> - o <counts/>

Where answers.dab is the name of your answers file, dabs/ is the name of the directory that contains your dataset .dab and quant files, and counts/ is the name of the output directory. This will produce a global.txt counts file in the /counts directory.

*Note*: If you are generating Bayes nets with multiple context-specific gene lists (such as for developmental stages or tissue types), you’ll need to run this step for each context. To generate counts for each context, add the context file name to the end of the command line:

Counter –w <answers.dab> -d <input_data/> - o <counts/> <context_gene_list.txt>

This will produce a context counts file (with the same name as the context gene list file) in the output directory.

Open the global.txt counts file to verify it looks correct. These files should contain counts of positive and negative values in each bin for each dataset in the input directory. Here’s a example of the first several rows of a global counts file that references the total number of datasets, the number of negative and positive genes, and counts for each dataset (only 3 of a total of 65 dataset counts are shown):

global 65

18239 1709

dataset1

448 1870 3230 1723 409 69 16

114 352 556 352 104 20 8

dataset2

261 1041 1773 987 209 46 15

74 244 409 246 65 16 11

dataset3

118 496 773 531 96 16 5

65 254 372 229 59 8 2

Note that if you used a global gene list for your context file, your global and context count files should be the same.

For more information on Counter, see <http://huttenhower.sph.harvard.edu/sleipnir/Counter.html>.

Produce a dataset list

Use the global counts file to create a numbered list of datasets in the order in which Counter processed them. This should be a tab-delimited text file that contains the sequence number and the data file name (with no extension). For example,

1 <tab> dataset1

2 <tab> dataset2

3 <tab> dataset3

4 <tab> dataset4

Save this dataset file to your network directory. You may find it easier to write a script to create a numbered dataset file using the global.txt output file from the first Counter step. (If you are using multiple contexts, you will need to create a context.con file that contains a numbered list of all context files to be used when generating the Bayes net. A context.con file is a tab-delimited text file that contains one line in the following format:

1 <tab> context1_gene_list <tab> context1_gene_list

2 <tab> context2_gene_list <tab> context2_gene_list

3 <tab> context3_gene_list <tab> context3_gene_list

Save the context.con text file to your gene list subdirectory.

Generate CPTs (Counter –k Step)

Use **Counter** to populate CPTs that comprise the naïve Bayesian network using the counts files and the dataset list created above. This step will produce a networks.bin output file that contains the Bayes net CPTs.

To generate CPTs, use the **Counter** -k command:

Counter –k <counts/> -o <networks.bin> -s <datasets.txt> -b <counts/global.txt>

Where counts/ is the name of the output directory you specified in the Counter –w step, networks.bin is the name of the Bayes Net output file, counts/global.txt is the path and file name of the global counts file created the –w step.

The Counter –k command will produce a networks.bin output file that contains CPTs.

*Optional Regularization Parameter*

If you are performing regularization, append a pseudocount parameter to the Counter –k command:

-p <pseudocount>

Where <pseudocount> is the regularization parameter used to modulate the strength of regularization required as implied by the strength of the prior (higher pseudocount values weaken the influence of redundant datasets.) You will need to test several pseudocount values (e.g., 1, 5, 10, 20, 30, …) to determine the optimal regularization strength for your data compendium.

*Optional Context parameter*

If you are using contexts, append a context parameter to the Counter –k command:

-X <context.con>

Where <context.con> is the text file that contains the lists of context files. (Note that the context file switch is a capital X.)

Make predictions (Counter –n Step)

The **Counter** –n command performs Bayesian inference for the classifier saved in the networks.bin file created as output of the Counter –K step. It produces a predictive functional relationship network graph file. If no context files are specified, it will generate a single global.dab file containing edges for all possible gene pairs and inference scores (edge weight) for each edge.

To infer functional relationships based on the CPT tables generated in the –k step, use the **Counter** –n command:

Counter –n <networks.bin> -o <results/> -d <dabs/> -s <datasets.txt> -e <gene_list_numbered.txt>

Where networks.bin is the name of the Bayes Net output file created in the –k step, results/ is the name of the output directory, dabs/ is the name of the data directory that contains .dab and quant files, datasets.txt is the name of the dataset list, and gene_list.txt is a numbered list of all protein coding genes. If you are using one or more contexts files, append them to the end of this command line.

If you are using contexts, append the name of your context file to this command (after the –e switch) and Counter will create a .dab file for each context.

*Additional Option for Regularization*

If you are regularizing your data, you will need to append a mutual information alpha file parameter to the Counter –n command:

-a <dataset_weights.dat>

Where <dataset_weight.dat> is the alpha file that summarizes the mutual information scores for each dataset in the data compendium. To generate this alpha file, use MIer to create a mutual information (MI) file for each dataset in your dabs/ directory, create an MI matrix using Combine_to_matrix.rb, and scale the raw scores with half2weights.rb. The basic commands are:

MIer -y 0 <dataset.dab> dabs/*.dab > dataset.dab.mi
Ruby combine_to_matrix <path_to_MI_files>
ruby half2weights.rb <bn_xdsl file) < <matrix.dat> > outfile

The Ruby scripts referenced are available on the Sleipnir site. To generate the xdsl file, convert the networks.bin file to an xdsl file using Sleipnir BNs2Text.

### Analyze Bayes Net Performance

Use DChecker to produce information needed to generate statistics required to analyze performance. To run DChecker, use the command:

DChecker –w <answer.dab> -i <results/global.dab> -g <gene_list.txt> > <performance_results.txt>

Where <answers.dab> is the gold standard answer file specified in the –w step, results/global is the path and file name of the network output file generated in the –n step, gene_list.txt is a list of all protein coding genes, and performance_results.txt is the name of the output file that will capture DChecker information. This will produce counts of True Positives, True Negatives, False Positives, and False Negatives for 1000 cuts and an AUC score (for the Area Under Curve). Version 3.0 of Sleipnir DChecker also includes columns for Precision (PR) and Recall (RC). If you use these calculated values, note that the column labels are transposed (as of the time of publication). For finer control over analysis, you can use a –b switch to specify the exact number of bins for quantile sorting by increasing the number of cuts from 1000 to 10,000 or 20,000, depending on the total number of genes in your gene list and the amount of detail you want in your performance analysis. For example if you have ~20,000 genes in your master gene list, and want to specif7 one bin per gene, use this command:

DChecker –w <answers.dab> -i <results/global.dab> -g <gene_list.txt> -b <20000> > <performance_results.txt>

Use the output file counts to calculate performance measurements of Precision, Recall, Sensitivity or True Positive Rate (TPR), and 1-Specificity or False Positive Rate (FPR):

Precision TP/(TP+FP)

Recall TP/(TP+FN)

TPR TP/(TP+FN)

FPR FP/(FP+TN)

Use R or Excel to produce a Receiver Operating Characteristic (ROC) curve, which is a graphical plot of TPR versus FPR. The AUC score reported in the final row of the DChecker outfile is the area under the ROC curve. You should also produce a plot of Precision versus Recall (use logarithmic scale for Recall on the X-axis), and other performance metrics as warranted.

The ROC and PR curves are only two performance metrics you can use to analyze network performance, but they are a good starting point. If you have questions, please contact the StemSight team at [stemsight@jax.org](mailto:stemsight@jax.org). Good luck with your networks!

For more information about DChecker options, see <http://huttenhower.sph.harvard.edu/sleipnir/DChecker.html>.

For more information about performance statistics for evaluating naïve Bayes Nets, see <http://en.wikipedia.org/wiki/Receiver_operating_characteristic>.

For more information about StemSight Bayes Nets from the Hibbs Lab, see http://stemsight.org.
